# The Use of Mass Spectrometric Techniques to Differentiate Isobaric and Isomeric Flavonoid Conjugates from *Axyris amaranthoides*

**DOI:** 10.3390/molecules21091229

**Published:** 2016-09-19

**Authors:** Łukasz Marczak, Paulina Znajdek-Awiżeń, Wiesława Bylka

**Affiliations:** 1 European Centre for Bioinformatics and Genomics, Institute of Bioorganic Chemistry, Polish Academy of Sciences, Noskowskiego 12/14, 61-704 Poznan, Poland; 2 Department of Pharmacognosy, Poznan University of Medical Sciences, Świecickiego 4, 60-781 Poznan, Poland; paulina.znajdek@gmail.com (P.Z.-A.); wieslawabylka@o2.pl (W.B.)

**Keywords:** collision-induced dissociation, electrospray, flavonoid conjugates, *Axyris amaranthoides*, tandem mass spectrometry, fragmentation spectra, isomeric compounds, isobaric compounds

## Abstract

Flavonoids are a group of compounds that are commonly found in various plants, where they play important roles in many processes, including free radical scavenging and UV protection. These compounds can also act as chemical messengers, physiological regulators or protectants against pathogens in the defense reactions of plants. Flavonoid activity is regulated by the addition of various substituents, usually mono- or oligosaccharides of common sugars, such as glucose, rhamnose or galactose. In some plants, glucuronic acid is attached, and this sugar is often acylated by phenylpropanoic acids. Identification of these compounds and their derivatives is of great importance to understanding their role in plant metabolism and defense mechanisms; this research is important because flavonoids are frequently a significant constituent of the human diet. In this study, we identify the flavonoid conjugates present in *Axyris amaranthoides* L. extracts and demonstrate the usefulness of high-resolution mass spectrometry (HRMS) analyzers for the differentiation of isobaric compounds and the utility of fragmentation spectra for the differentiation of isomeric structures. According to our knowledge, some of the structures, especially dehydrodiferulated conjugates of tricin, whose structures are proposed here have been found for the first time in plant material.

## 1. Introduction

Flavonoids are present in plants mainly as glycosides; glucuronides, in which uronic acids occur as sugar units, are less frequent [1]. The role of glucuronides in plants has not been yet clarified. It is speculated that they can provide protection against herbivores [2]. Flavonoid glucuronidation can also be catalyzed by enzymatic reactions in human and animal metabolic pathways [3].

The main flavonoid glucuronides identified thus far in plants include glucuronoflavones, especially apigenin and luteolin, as well as tricin, chrysoeriol, scutellarein and the glucuronoflavonols quercetin, kaempferol, isorhamnetin and myricetin [4]. These constituents have been reported in Asteraceae (*Cirsium rivulare*, *Erigeron multiradiatus*, *Tanacetum parthenium*) [5,6,7], Polygonaceae (*Polygonum amphibium*, *P. aviculare*) [1,8], Lamiaceae (*Scutellaria indica*, *S. baicalensis*, *Salvia officinalis*, *S. palestina* [9,10,11,12], Scrophulariaceae (*Picria fel-terrae*) [13], Pedaliaceae (*Uncarina* sp.) [14] and Chenopodiaceae (*Spinacia oleracea*) [15]. Diglucuronoflavones identified in a few species from Fabaceae (*Medicago truncatula*, *M. radiata*, *M. sativa*) [16,17,18,19], Lamiaceae (*Perilla ocimoides*) [20], Poaceae (*Secale cereale*) [21] and Hydrocharitaceae (*Elodea canadensis*) [22] are particularly rare. In *Medicago* sp., isolated diglucuronoflavones were additionally acylated with the hydroxycinnamic acids caffeic, ferulic, sinapic and *p*-coumaric acids [17,18,19].

Some glucoronidic derivatives of flavonoids have been shown to exert antioxidant and anti-inflammatory activities [8,23]. In in vitro studies, quercetin 3-*O*-glucuronide prevented vascular smooth muscle cell hypertrophy by angiotensin II. Moreover, flavonol glucuronides from *Polygonum amphibium* L. induced apoptosis in HL-60 and Jurkat cells [1]. A study conducted in rabbits demonstrated that a flavonoid glucuronide fraction isolated from *Tulipa gesneriana* exerted microvascular protective effects against skin vascular permeability induced by chloroform or histamine [24].

*Axyris amaranthoides* L. (*Chenopodiaceae*) is a herbaceous annual plant (20–80 cm in height), commonly known as Russian Pigweed. This plant originated in Asia but is naturally widespread throughout the USA. *A. amaranthoides* herb is used in Chinese folk medicine as a gastrointestinal spasmolytic and to regenerate the liver, as well as alleviating rheumatic pain and swelling [25,26].

Utilization of liquid chromatography and mass spectrometry in analysis of phenolic compounds, especially flavonoids, is extensively studied in the literature and the use of different mass spectrometric analyzers and ionization techniques have grown to be the most common method in this type of studies [27,28]. High resolution tandem mass spectrometry (MS/MS) systems are capable to deliver precise information on molecular weight of compounds, and collision induced dissociation (CID) fragmentation used together with soft ionization techniques provides additional structural information [29]. Moreover, analysis of samples in both positive and negative ionization modes may support the study due to different fragmentation patterns of protonated and deprotonated molecules [30]. Nevertheless, all this information is not sufficient for complete structural elucidation of studied compounds due to almost identical fragmentation pathways of positional isomers. This situation is in accordance with flavonoid derivative fragmentation, where sugar moieties can be substituted at different positions of aglycones; moreover, these sugar moieties are often also acylated with organic acids at different hydroxyl groups [27]. There are some proposals in literature trying to find a method to distinguish differentially substituted isomeric flavonoid molecules. One of them is measuring relative abundances of specific fragmentation ions [31], and the other is electrospray ionization (ESI) analysis of metal complexes [32]. Such approaches are interesting but do not allow unambiguous identification and depend strongly on analysis parameters and interpretation skills. The only confident way for complex structural analysis is still the use of different complementary spectroscopic methods for elucidation of molecule structure. In most cases, information derived from NMR and MS analysis leads to unambiguous structure elucidation [17,18]. Such approaches are possible only when compound of interest is present in relatively high concentrations and can be purified from the complex mixtures. Another way to confirm proper compound identification is the use of purified or commercially available standards. This way, it is possible to compare retention times of standards with potentially identified compound as well as comparison of their MS and multiple MS/MS (MSn) spectra. This approach is also commonly used in combination with ultraviolet diode array detectors (UV-DAD) [33]. High resolution MS was used to identify saponins and flavonoids in *Camellia oleifera* [34], using a ultra high performance liquid chromatography coupled with electrospray ionization hybrid linear trap quadrupole orbitrap mass spectrometry (UHPLC-LTQ-orbitrap-MSn) system, it was possible to tentatively characterize seven flavonol glycosides, and MS^3^ spectra were used for unambiguous aglycone characterization. Another interesting work showing usefulness of the high performance liquid chromatography coupled with tandem mass spectrometry (HPLC-MSn) approach for comprehensive plant metabolites characterization was published by Wang and co-workers [35], where authors propose general strategies for analysis of complex samples. Application of such a strategy allowed temporary identification of 48 flavonoid derivatives among 144 compounds in total, while NMR analysis was further used for structures confirmation of some novel compounds.

Based on a literature search, *A. amaranthoides* has been the subject of just several phytochemical studies. Extracts of *A. amaranthoides* roots and seeds were found to contain large amounts of phytoecdysteroids, and 1α,20*R*-dihydroxyecdysone, 20*E*-hydroxyecdysone (polypodine A) and 5,20*E*-dihydroxyecdysone (polypodine B) have been isolated [36,37]. There is no further information in the available literature on flavonoid content of *A. amaranthoides* that we could confront with our results. This facts are making analysis on MS systems more difficult due to lack of information on general glycosylation pattern of aglycones provided by enzymatic systems responsible for acylation and glucosidation of flavonoids specifically in *A. amaranthoides*. Therefore, all proposed structures in this paper should be treated as tentative and are based on the most common substitution patterns found in available literature.

In this study, we demonstrate that *A. amaranthoides* herb is a rich source of flavonoid compounds, mainly tricin acylated glucuronides, which are quite rare in plants. To the best of our knowledge, some of the structures proposed here have been shown for the first time in plants.

## 2. Results and Discussion

### 2.1. Flavonoid Glycosides of Axyris amaranthoides

Due to the presence in examined fractions of a large number of flavonoids of similar structures and polarities, the chemical constituents in the mixture were identified using HPLC combined with high-resolution tandem quadrupole-time of flight (QToF) mass spectrometry. A detailed analysis of the MS and MS^2^ spectra led to the successful characterization of 35 flavonoid conjugates, including previously identified compounds [19] and previously unknown flavonoid derivatives.

**Figure 1:**
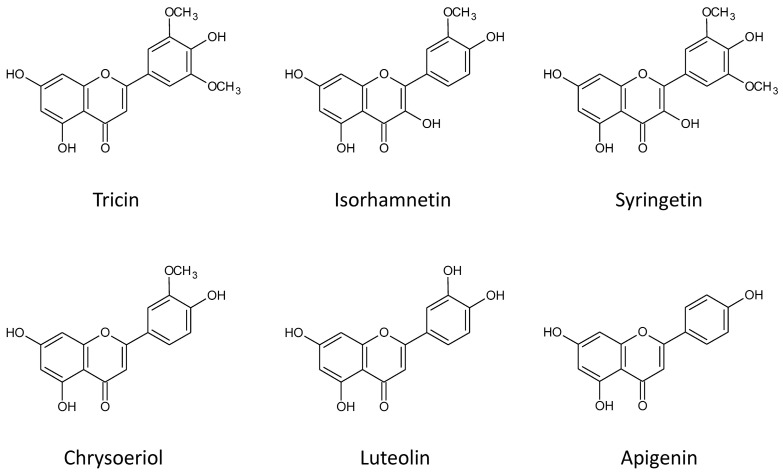
Structures of flavonoid aglycones identified in *Axyris amaranthoides*.

All of the tentatively identified compounds are presented in Table 1. Most of them were tricin derivatives (18 compounds). Others compounds included derivatives of chrysoeriol (seven compounds), isorhamnetin (five compounds), apigenin (two compounds), luteolin (one compound) and syringetin (one compound). Assigning the correct structures for aglycones was possible due to pseudo MS^3^ analysis and comparison of obtained fragmentation spectra with spectra of standards and spectra found in literature or deposited in common spectral libraries. This work was done as described in our earlier papers [19,38]. Structures of identified aglycones are presented in Figure 1, and fragmentation spectra and proposed structures of the conjugates are shown in supplementary data (Figures S1–S35). The conjugates included both acylated and non-acylated flavonoid glycosides with attached glucose, xylose and/or glucuronic acid moieties. Most of the recognized metabolites were acylated with various phenolic acids, 13 metabolites were feruloylated, nine metabolites were acylated with *p*-coumaric acid, and one metabolite was acylated with sinapic acid. Additionally, we propose that two of the metabolites were acylated with dehydrodiferulic acid, and one was acylated with benzoic acid.

Because we exclusively used MS techniques to identify the mixture components, we were unable to assign the positions of particular moieties in aglycone molecules, and we were also unable to distinguish the exact sugar substituents. Based only on our experience and the available literature, we propose tentative structures of identified compounds. We are aware that analysis of standards should be done to perform correct confirmation of our statements; nevertheless, we dealt with complex mixtures of compounds often found in very low concentrations, and additional separation of specific compounds or even chemical synthesis would be impossible or very complicated and time-consuming. Many studies have described the typical substitution patterns of flavonoids. NMR studies have identified the phenylpropenoic acidic moieties (*p*-coumaric, ferulic and sinapic acids) of these conjugates, and the positions of the ester bonds and the glycosidic bonds between the aglycone and the sugar moiety(ies) have been established [16]. In addition, reports of similar structures have been confirmed in various species [17,18]. It should be noted here that, thanks to use of the high resolution mass spectrometry (HRMS) system, we were able to distinguish isobaric fragment losses of ferulate moiety from glucuronate moiety (loss of 176.046 Da to 176.031 Da), and coumarate from rhamnose moiety (loss of 146.036 Da to 146.057 Da). Additionally, these isobaric substituents can be differentiated based on comparison of positive and negative ion fragmentation spectra, since ferulate gives a characteristic neutral loss of 194 Da and coumarate gives a loss of 164 Da. Based on these findings, we propose certain structures for the flavone derivatives found in *Axyris amaranthoides* with the reservation that these structures were not determined directly using analytical methods that permit unambiguous determination.

**Figure 2:**
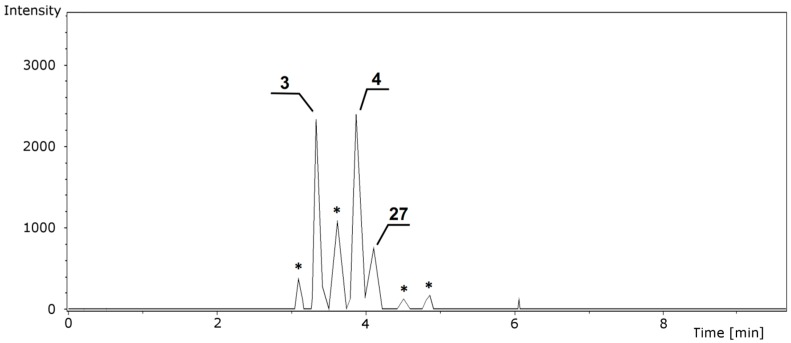
Extracted ion chromatogram at *m*/*z* 815 showing all isomeric and isobaric ions. Asterisks indicate isomeric compounds not possible to distinguish due to very similar spectra to compounds **3**, **4** or **27**.

**Figure 3:**
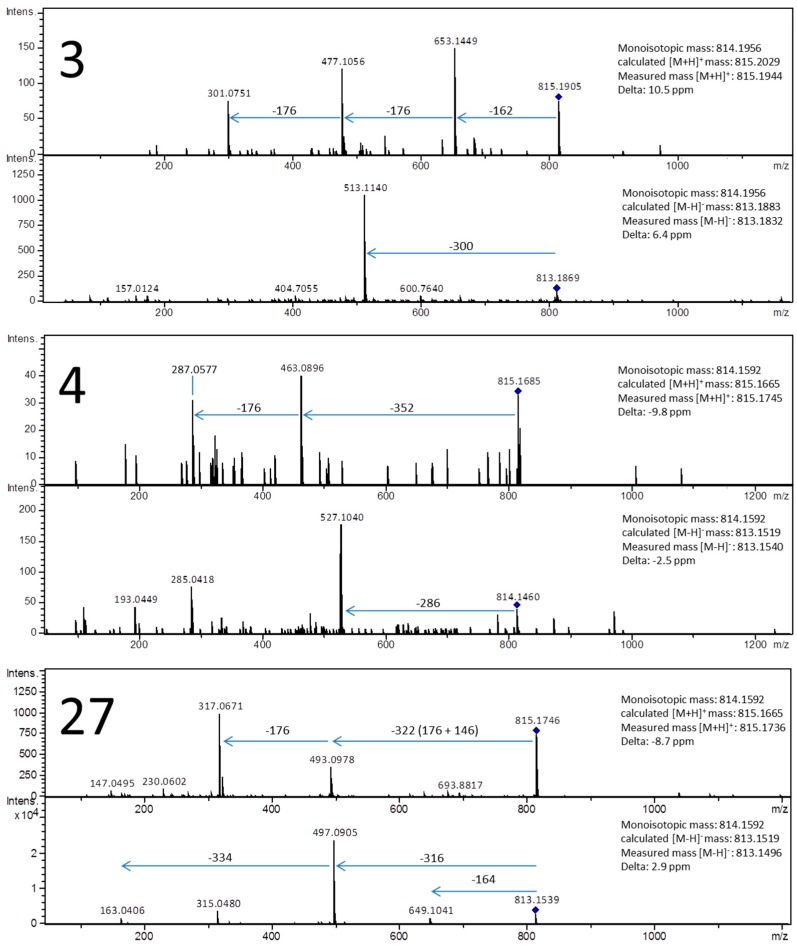
Mass spectra registered in positive and negative ion mode for isomeric and isobaric compounds: chrysoeriol 7-*O*-[2-*O*-feruloyl-glucuronopyranosyl-(1-3)-*O*-glucopyranoside] (**3**), luteolin 7-*O*-[2′-*O*-feruloyl-glucuronopyranosyl-(1-2)-*O*-glucuronopyranoside] (**4**) and isorhamnetin 3-*O*-[2′-*O*-coumaroyl-glucuronopyranosyl-(1-2)-*O*-glucuronopyranoside] (**27**).

In this study, all samples were analyzed in both positive and negative ion modes, spectra were acquired using a high-resolution q-ToF mass spectrometer that enabled precise measurements of *m*/*z* values for all [M + H]^+^ and [M − H]^−^ ions in both MS and MS^2^ modes. Because we have previously discussed the necessity for high-resolution MS systems [19], here we will only emphasize the inability of low-resolution MS analyzers such as quadrupole or ion trap instruments to determine isobaric compounds or isobaric fragment ions. Furthermore, knowing the exact mass of protonated or deprotonated ions and having precisely calibrated the system prior to analysis enhances the ability of these methods to correctly determine structures. Finally, the use of a UPLC system greatly affected the acquisition of the data; chromatographic resolution was an important factor in the analysis due to the close structural similarities of some of the flavone glucuronides. It is notable that the resolving power of UPLC systems enabled the studied compounds to be separated within 8 min during a 12-min chromatographic run. Hyphenation of this system with a fast high-resolution mass spectrometer allowed the acquisition of MS^2^ spectra for consecutive compounds. Figure 2 shows the extracted ion chromatogram (EIC) for the product ion at *m*/*z* 815; seven peaks were observed, three of which were successfully characterized based on their exact mass and fragmentation spectra (Figure 3).

Each of the compounds has a different aglycone, i.e., chrysoeriol (**3**), luteolin (**4**) and isorhamnetin (**27**). Furthermore, compounds **4** and **27** are isomers. In addition to containing different aglycones, these two conjugates are acylated differently. Compound **4** is conjugated with ferulic acid, and compound **27** is conjugated with coumaric acid; thus, these two compounds have the same molecular formula. These acylating moieties were differentiated using positive and negative ion mode fragmentation spectra. The mass of compound **3** differs from those of compounds **4** and **27** by approximately 40 mDa, which is too large of a difference to be attributed to improper calibration; we propose it has a different molecular formula so that these results suggest that compound **3** is isobaric with compounds **4** and **27** (see Table 1). Again, analysis of positive and negative spectra leads to unambiguous identification of the substituents in compound **3**. Other isomeric compounds were differentiated in our study by analyzing fragmentation spectra, including the following compound pairs: **29** and **30**, **18** and **20**, **10** and **23**, **11** and **25**, **13** and **15**, and **14** and **21**. Other pairs of isobaric compounds that were effectively recognized in extracts of *Axyris* *amaranthoides* are **1** and **22**, and **6** and **12**.

### 2.2. Fragmentation Pathways of Flavone Glycoconjugates

Only eight out of the 35 compounds detected in *Axyris amaranthoides* were non-acylated flavone mono- and diglycosides. These compounds contained linear saccharides, which were attached most probably at position 7 of chrysoeriol and tricin. Based on previously reported work [39,40], the most probable site of glycosylation is position 3 of isorhamnetin, but based only on MS/MS spectra, we can not exclude the possibility that the saccharides are attached at position 7 as well. Compounds **8**, **16** and **28** were mono-glucuronates of isorhamnetin, tricin and chrysoeriol, respectively. The glycosidic moiety of compounds **1**, **2** and **5** comprised two combined glucuronic acid molecules, and compound **22** was glucuronosylo-glucoside but with two sugar moieties substituted at different positions. Compound **26** contained one methyl ester of glucuronic acid.

CID fragmentation spectra of these compounds, which were acquired in positive ion mode, included only product ions that were created after successive cleavages of glycosidic bonds between sugars and between the sugar moiety and the aglycone (see Supplementary Materials). A positive charge was retained on the aglycone, and an entire series of Y_n_^+^ product ions were observed [41,42]. This fragmentation pattern is equivalent to that observed earlier by us [19] and to patterns observed by others for all flavonoid glycosides [42].

The fragmentation pathway observed in the negative ion mode (deprotonation) was different from the fragmentation pathway observed in the positive ion mode. As in our previous work [19,27], we observed a predominant loss of aglycones in the case of diglycosides, and the charge was retained on the sugar moiety, representing the most abundant peak in the spectrum. For diglucuronides, we observed an ion at *m*/*z* 351, which is typical of these compounds, as described in our previous study [19]. Interestingly, for monoglucosides, the fragmentation pathway observed in the negative ion mode is similar to that observed with positive spectra; namely, we observed a loss of sugar and charge retention on the aglycone, which suggests that only di- and triglucuronate moieties can efficiently stabilize the negative charge. This finding is consistent with other reports [43]. In the positive ion mode, a loss of hexose was observed from compound **22**, followed by the loss of glucuronic acid; however, in the negative ion mode, the opposite was true, i.e., glucuronic acid was lost before hexose. This clearly indicates that the two substituents are directly attached to the aglycone in different positions; therefore, it is not possible that these two sugar moieties are linked to each other. In this case, analysis of mass spectra does not give the information on exact position of glucuronic acid with respect to glucose moiety.

The glycosylated flavone tricin 7-*O*-[glucuronopyranosyl-(1-2)-*O*-methylglucuronopyranoside] (compound **26**) was also recognized. When compared to compound **5**, which is a non-methylated tricin diglucuronide, we observed a completely different fragmentation scheme. In the methylated diglucuronide, charge is retained on an aglycone fragment in the negative mode; therefore, MS/MS spectra recorded for **26** using the positive and negative ion modes appear very similar. This demonstrates that although two carboxyl groups in the diglucuronate moiety strongly stabilize the product ion [19], blocking one of these carboxyl groups dramatically alters the stability of this negative ion, resulting in charge retention on the aglycone. Few studies have analyzed flavone diglucuronides using negative-ion MS/MS, although many quercetin and methylquercetin diglucuronides have been isolated from animal tissues or body fluids [44,45]. In these reports, no product ion at *m*/*z* 351 was reported, suggesting either that quercetin glucuronides act differently or that both sugars were attached to different hydroxyl groups on the aglycone. However, the cited authors did not address this issue.

### 2.3. Fragmentation Pathways of Acylated Flavone Glycoconjugates

Among the compounds found in this work, 27 were acylated with various phenolic acids, mainly ferulic acid (16 compounds) and *p*-coumaric acid (nine compounds), and with sinapic, benzoic and hydroxyferulic acids (one compound each). In addition, we found two tricin conjugates, which we propose are acylated with dehydrodiferulic acids. All of the characterized flavonoid conjugates contained at least two sugar moieties, and some contained three. We detected no acylated monoglycosylated flavones, consistent with our previous report [19] and those of others [16,17,18].

Analysis of the fragmentation pathways of acylated flavones showed that they behave similarly to non-acylated compounds, especially in the positive ion mode, for which we observed a sequential loss of consecutive substituents that left the aglycone ion as a final fragment. Apparently, terminal phenolic acid groups always leave the pseudomolecular ion together with the sugar units to which they are connected; consequently, we observed characteristic losses of 352 Da for ferulate and 322 Da for coumarate. The same effect was observed with hydroxyferulate (368 Da) and benzoate (280 Da). However, because only one of each type of these compounds (**23** and **32**) was observed in our experiment, we cannot be certain that this result is common in all such cases.

When phenylpropenoic acid units were attached to non-terminal sugar moieties, we observed different fragmentation behavior: for compounds **7**, **9**, **11**, **13**, **14** and **15**, in which the middle glucuronates were acylated, we observed the loss of a single sugar unit (in all cases, this sugar was a hexose, presumably glucose (162 Da)). Subsequently, an acylated glucuronide fragment was removed from the resulting protonated residue, and the final fragmentation step was cleavage of the *O*-glycosidic bond between glucuronic acid and the aglycone. For compound **21**, we assigned the acylation position to the glucuronate unit that was directly attached to tricin. The initial fragmentation step was the elimination of a glucuronate-glucose moiety (352 Da); this step was followed by the loss of a ferulate unit. Surprisingly, in the final fragmentation step, a charge was retained on the glucuronic acid residue, which is not typical of positive ion mode fragmentation.

**Figure 4:**
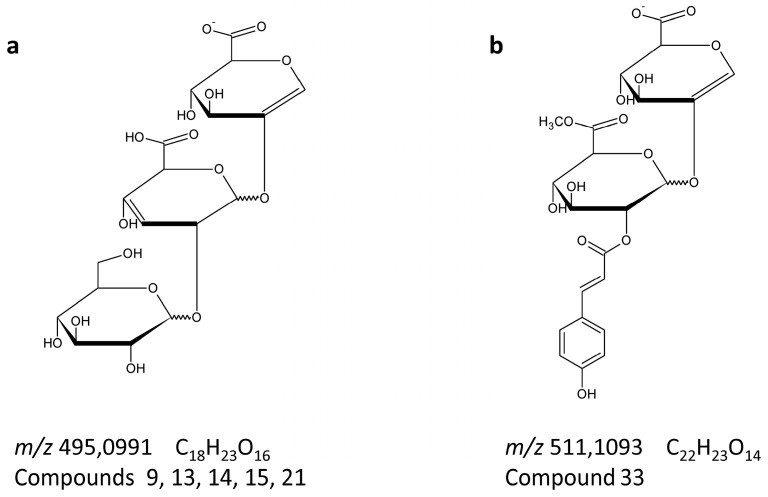
Structures of the characteristic product ions of flavonoid glucuronates, (**a**) *m*/*z* 495 found in acylated at middle sugar moiety compounds, with linear fragment comprising two units of glucuronic acid and one unit of glucose; and (**b**) *m*/*z* 511 corresponding to methylated diglucuronate acylated with *p*-coumaric acid. Ions were found in the collision induced dissociation fragmentation (CID MS/MS) spectra, registered in negative mode, referred to the compounds from Table 1 and spectra in supplementary data.

The fragmentation spectra of acylated glycoconjugates acquired in the negative ion mode were interesting; we observed common fragment ions having characteristic masses of *m*/*z* 497, *m*/*z* 527 and *m*/*z* 513. These masses correspond to acylated sugar moieties comprising two molecules of glucuronic acid or glucuronic acid conjugated to a glucose unit. We have previously described fragmentation pathways leading to these fragments [19]. Here, we report a further characteristic ion at *m*/*z* 495, which corresponds to a linear fragment comprising two units of glucuronic acid and one unit of glucose (Figure 4a). This fragment was detected in the spectra obtained for compounds **9**, **13**, **14**, **15** and **21** and appears to be present only when sugar rings are connected by glycosidic bonds in positions 1 → 2. We did not observe this ion in compounds **7** and **11**, which also contain linear triglucosides, but instead observed a glycosidic bond in positions 1 → 3 between the two units (see the Supplementary Materials). Another observed ion that was not previously reported is a fragment of *m*/*z* 511 that was observed in the negative ion spectrum of compound **33**; we assigned this result to a fragment that originated from *p*-coumaric acid and two units of glucuronic acid, in which one of these units contained a methylated carboxyl group (Figure 4b).

As was demonstrated above by the results obtained for compound **22**, the analysis of spectra that were acquired in both positive and negative ion modes resulted in the tentative identification of structures with sugar moieties substituted at two positions. This was also observed with the acylated compounds **24** and **25**. In the positive ion spectrum, we observed two possible losses occurring from the pseudomolecular ion: the loss of a fragment (132 Da) corresponding to a pentose molecule (presumably xylose) and a second possible cleavage that can be attributed to the loss of coumaroyl glucuronide (322 Da). Further fragmentation steps form a deprotonated chrysoeriol molecule. In addition, in the negative ion spectrum, we observed the loss of a neutral pentose fragment, followed by cleavage of an aglycone, to a characteristic ion at *m*/*z* 497, representing a deprotonated coumaroyl diglucuronide [19]. The same fragmentation pathways were observed with compound **25** except that a different aglycone was present—in this case, tricin. It should be noted here that it was not possible to define the position of xylose substituent based only on the mass spectra, but we can presume that, due to steric hindrance, the most possible site for xylose group in compounds **24** and **25** is hydroxyl group at 4′ position.

In this work, we detected fine fragmentation spectra of precursors with relatively high molecular masses exceeding 1000 Da; i.e., compounds **34** and **35**, with masses of 1036 Da and 1052 Da, respectively. Analysis of the fragmentation pathways led to the conclusion that these compounds were tricin glycoconjugates that were acylated with dehydrodiferulic acids; to the best of our knowledge, these flavone derivatives are reported for the first time. We observed that the acylating group in compound **35** is cyclobutane diferulic acid [46,47]. This ferulic acid dimer is formed by cyclodimerization in plant exposed to UV irradiation [48]. Therefore, we cannot be certain if this compound is native to *Axyris amaranthoides* or if it was produced during sample preparation when the samples were subjected to UV rays from daylight irradiation. This compound has a characteristic fragmentation pattern in the positive ion mode. In the initial fragmentation step, one molecule of ferulic acid is detached from its dimer; consequently, an ion at *m*/*z* 859 is created that corresponds to a molecule containing ferulated diglucuronide (compound **19**). Further fragmentation steps are identical to those observed with compound **19**. In the negative ion mode, we observed a classic loss of aglycone, followed by the consecutive loss of two glucuronide moieties, resulting in the formation of an ion (*m*/*z* 371) that corresponds to a deprotonated cyclobutane dimer of ferulic acid. Finally, a fragment at *m*/*z* 193 is present corresponding to single, deprotonated molecule of ferulic acid.

**Figure 5:**
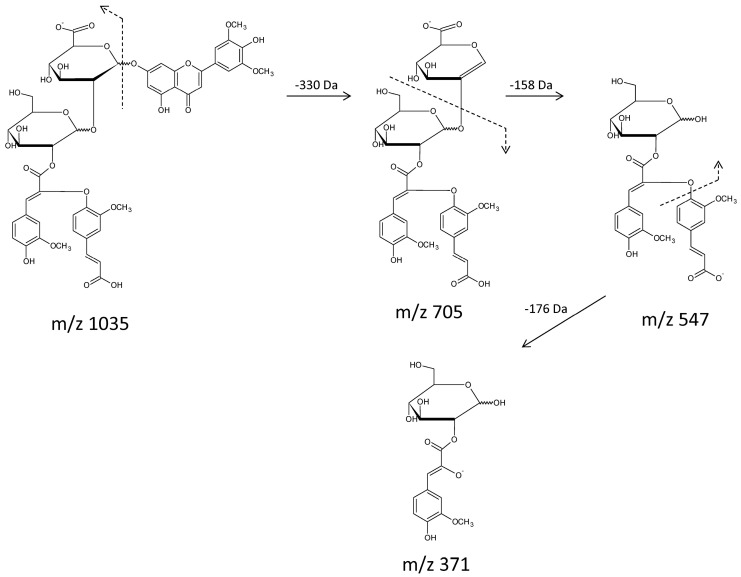
Proposed fragmentation pathway for compound 34 based on MS/MS spectrum registered in negative ion mode.

Compound **34** contains non-cyclic dehydrodiferulic acid, which is frequently found in plant cell walls, especially in grasses, where it serves as a chemical link between chains of arabinoxylans, among others [47,49]. Such dimers are produced by enzymatic reactions involving oxidative enzymes such as peroxidase; thus, it is likely that compound **34** is a native constituent of these plants. Dehydrodiferulates occur naturally in many isomeric forms [50]; therefore, we cannot conclude which isoform is present in our finding. The fragmentation behavior of these components is not typical and is different from that observed for compound **35**. Here, in positive mode, we detected the loss of a 506 Da fragment corresponding to aglycone glucuronide (330 Da for tricin and 176 Da for glucuronic acid). Charge is retained on dehydrodiferuloyl glucoside (*m*/*z* 531), although the aglycone ion itself remains present, and this ion is the second most abundant ion in the spectrum. The fragmentation pathway obtained by analyzing the negative ion mode spectrum provides supplementary information regarding the identified compound. In the initial fragmentation step, we observed a typical loss of aglycone, followed by the loss of glucuronic acid; then, dehydrodiferulate is cleaved, followed by the loss of ferulic acid (176 Da). Consequently, a [M − H]^−^ ion with *m*/*z* 371 is observed, which corresponds to a glucose α-hydroxyferulate fragment. The proposed fragmentation scheme is presented in Figure 5.

## 3. Experimental Section

### 3.1. General

Thin-layer chromatography (TLC) was performed on cellulose plates (Merck, Darmstadt, Germany) using 1% KOH in MeOH for detection. The plates were irradiated with UV light at 366 nm before and after spraying.

Column chromatography was performed using Sephadex LH-20 (Pharmacia Biotech AB, Uppsala, Sweden) and cellulose powder Whatman CF 11 (Whatman International Ltd., Maidstone, Kent, UK). The solvent systems used were as follows: S1: (EtOAc–MeOH–H_2_O 100:6:6); S2: (EtOAc–MeOH–H_2_O 100:10:9.5); S3: (EtOAc–MeOH–H_2_O: 100:16:13) and S3: (EtOAc–MeOH–H_2_O: 100:18:15).

Solid-phase extraction (SPE) was performed using a System BAKER SPE 12-G) equipped with a Bond Elut C18 cartridge (Agilent Technologies, Palo Alto, CA, USA). Methanol, ethyl acetate and KOH were obtained from POCh, Gliwice, Poland. All reagents and solvents used for chromatography were of analytical or spectrometric grade.

### 3.2. Plant Materials

*Axyris amaranthoides* herb was cultivated in the garden of the Department of Medical Plants at Poznan University of Medical Sciences. The seeds were obtained from Warsaw University Botanical Garden. The plant was collected in August 2012 during the flowering period and authenticated by MSc Katarzyna Guranowska from Warsaw University, Warszawa, Poland. Voucher specimens were deposited in the Department of Pharmacognosy, Poznan University of Medical Sciences, Poznan, Poland (AX 253).

### 3.3. Extraction of Phenolic Compounds

Air-dried *A. amaranthoides* herb (300 g) was successively extracted with MeOH and 50% MeOH. The combined extracts were evaporated to dryness, and the dry extract was treated with hot H_2_O, filtered and then partitioned between CHCl_3_ and EtOAc. The concentrated chloroform extract was loaded onto a Sephadex LH-20 column (Pharmacia Biotech AB, Uppsala, Sweden) and washed with 50% MeOH to yield a crude powder (10 mg). The ethyl acetate fraction was chromatographed on cellulose powder with S1 as the eluent to yield a crude product (12 mg), which was then purified by Sephadex LH-20 column chromatography and eluted with 50% MeOH. The aqueous residue after extraction with CHCl_3_ and EtOAc was separated on a cellulose column; fractions were eluted first with S2, then with S3 and finally, with S4, yielding 128 fractions. After TLC, control fractions 73–90 were combined and rechromatographed on Sephadex LH-20 using first H_2_O and then 50% MeOH to yield another product (30 mg). Final purification of the obtained products was achieved using solid-phase extraction (SPE) according to the Svedström method with minor modifications [51]. The cartridge was first conditioned with 10 mL of methanol and 5 mL of water; then, 5 mL of water was added to the dry compounds, and 1 mL of this solution was loaded onto the cartridge. Fractions were then eluted with water followed by mixtures of H_2_O:MeOH of increasing concentration from 5% MeOH to 40% MeOH.

### 3.4. Liquid Chromatography with Mass Spectrometric Detection

All fractions (see Section 3.3) were analyzed using an HPLC/MS system comprising a Waters Acquity UPLC system (Waters, Milford, MA, USA) combined with a Q-ToF mass spectrometer, model micrOTOF-q (Bruker Daltonics, Bremen, Germany). Analyses were carried out using Zorbax Eclipse XDB-C18 columns (2.1 mm × 150 mm, 3.5 µm and 2.1 mm × 100 mm, 1.8 µm; Agilent). Chromatographic separation using the Waters Acquity UPLC system was performed at a flow rate of 0.6 mL/min using mixtures of the following two solvents: A (99.5% H_2_O, 0.5% formic acid (*v*/*v*)); B (99.5% acetonitrile, 0.5% formic acid (*v*/*v*)). The column effluent was split 3:2 to allow 0.2 mL/min to be delivered to an ESI ion source. The elution steps were as follows: 0–8 min linear gradient from 5% to 30% B, 8–10 min linear gradient to 95% B, 10–12 min of isocratic flow at 95% B and a return to the initial condition after 3 min.

The micrOTOF-Q spectrometer consisted of ESI operating at ±4.5 kV, nebulization with nitrogen at 1.6 bar and a dry gas flow of 8.0 L/min at a temperature of 220 °C. For the pseudo MS3 analyses, additionally, in-source induced dissociation (ISCID) was set up at 80 eV to cleave *O*-glycosidic bonds in flavonoid conjugates enabling aglycone fragmentation spectra acquisition. The system was calibrated externally using a calibration mixture containing sodium formate clusters. Additional internal calibrations were performed for every run by injecting the calibration mixture using a divert valve during LC separation. All calculations were performed using the high precision calibration (HPC) quadratic algorithm. These calibrations yielded an accuracy of less than 10 ppm. MS/MS spectra were acquired at a frequency of one scan per second for ions that were chosen on the basis of preliminary MS experiments. The collision energy was dependent on the molecular masses of the compounds and was set between 10 and 25 eV. The instrument operated at a resolution higher than 10,000 (FWHM, full width at half maximum) using the program micrOTOF control version 2.3, and data were analyzed using the Data Analysis version 4 package, which was supplied by Bruker. Metabolite profiles were registered in positive and negative ion modes.

## 4. Conclusions

The use of an LC-MS/MS system comprising a rapid HPLC and a tandem MS/MS spectrometer equipped with a high-resolution time-of-flight analyzer permits the structural characterization of flavonoid glycoconjugates containing glucuronic acid and phenolic acyl substituents. However, due to the availability of complementary information resulting from differences in the fragmentation patterns for the positive and negative ions of these compounds, it is essential that analyses are performed using both modes of ionization. It is of particular importance that mechanisms observed for glycosides containing more than one molecule of glucuronic acid are dramatically different from those of flavonoid glycoconjugates containing sugars without carboxyl groups, especially when using the negative ion mode. Ions presented in Figure 4 may serve as diagnostic ones for correct recognition of presented here specific groups of flavonoid diglucuronides. HRMS analyses performed in both positive and negative ion modes might permit the structural characterization of glucuronyloglucosides and di- or triglucuronides. Such a system allows also for unambiguous differentiation between isomeric and isobaric compounds in opposite to low resolution MS. Additionally, we showed that, in MS/MS mode, differentiation of isobaric fragments (ferulate and glucuronate, coumarate and rhamnose, etc.) is also of great importance and is possible only on high-resolution MS systems. The use of modern analytical equipment might enable the characterization of potential new molecules as presented in this study, in which we demonstrate the acylation of flavone glucuronides with dehydrodiferulic acids. However, one should keep in mind that mass spectrometry itself does not allow for unambiguous identification of compounds and proposed structures should always be additionally confirmed using other methods, such as NMR spectroscopy. In our case, unfortunately, it was not possible due to the fact that these compounds were present in samples in very low amounts.

## Figures and Tables

**Table 1:** Flavonoid glycosides tentatively identified in *Axyris amaranthoides*.

#	Name	Elemental Composition	Exact Monoisotopic Mass (Da)	Fragment Ions in Positive Ion Mode (*m*/*z*)	Fragment Ions in Negative Ion Mode (*m*/*z*)
1	Isorhamnetin 3(*7*)-*O*-glucuronopyranosyl-(1-2)-*O*-glucuronopyranoside	C_28_H_28_O_19_	668.1224	669, 493, 317	667, 351, 315, 193, 175
2	Chrysoeriol 7-*O*-glucuronopyranosyl-(1-2)-*O*-glucuronopyranoside	C_28_H_28_O_18_	652.1275	653, 477, 301	651, 351, 299, 175
3	Chrysoeriol 7-*O*-[2-*O*-feruloyl-glucuronopyranosyl-(1-3)-*O*-glucopyranoside]	C_38_H_38_O_20_	814.1956	815, 653, 477	813, 513
4	Luteolin 7-*O*-[2′-*O*-feruloyl-glucuronopyranosyl-(1-2)-*O*-glucuronopyranoside]	C_37_H_34_O_21_	814.1592	815, 463,2 87	814, 527, 285, 193
5	Tricin 7-*O*-glucuronopyranosyl-(1-2)-*O*-glucuronopyranoside	C_29_H_30_O_19_	682.1381	683, 507, 331	681, 351, 193, 175
6	Tricin 7-*O*-[2-*O*-feruloyl-glucopyranosyl-(1-3)-*O*-glucuronopyranoside]	C_39_H_40_O_21_	844.2062	845, 683, 507	843, 513
7	Tricin 7-*O*-{2′-*O*-feruloyl-[glucopyranosyl-(1-3′)-*O*-glucopyranosyl]-(1-2)-*O*-glucuronopyranoside}	C_45_H_50_O_26_	1006.259	1007, 507	1005, 675, 513
8	Isorhamnetin 3(*7*)-*O*-glucuronopyranoside	C_22_H_20_O_13_	492.0904	493, 317	491, 315
9	Isorhamnetin 3(*7*)-*O*-{3′-*O*-coumaroyl-[glucuronopyranosyl-(1-2′)-*O*-glucopyranosyl]-(1-2)-*O*-glucuronopyranoside}	C_43_H_44_O_26_	976.212	977, 815, 493, 317	975, 811, 659, 513, 495, 337, 315
10	Syringetin 3-*O*-[2′-*O*-feuroyl-glucuronopyranosyl-(1-2)-*O*-glucuronopyranoside]	C_39_H_38_O_23_	874.1804	875, 523, 347	873, 527, 333, 193
11	Apigenin 7-*O*-{2′-*O*-feruloyl-[glucopyranosyl-(1-3′)]-*O*-glucuronopyranosyl-(1-2)-*O*-glucuronopyranoside}	C_43_H_44_O_25_	960.2171	961, 799, 447, 271	959, 689, 527, 193
12	Isorhamnetin 3(*7*)-*O*-[2′-*O*-feruloyl-glucuronopyranosyl-(1-2)-*O*-glucuronopyranoside]	C_38_H_36_O_22_	844.1698	845, 493, 317, 177	843, 649, 527, 315, 193
13	Chrysoeriol 7-*O*-{3′-*O*-feruloyl-[glucuronopyranosyl-(1-2′)]-*O*-glucopyranosyl-(1-2)-*O*-glucuronopyranoside}	C_44_H_46_O_26_	990.2277	991, 829, 477, 301	989, 689, 495
14	Tricin 7-*O*-{3′-*O*-feruloyl-[glucuronopyranosyl-(1-2)]-*O*-glucopyranosyl-(1-2)-*O*-glucuronopyranoside}	C_45_H_48_O_27_	1020.238	1021, 859, 507, 331	1019, 689, 495
15	Tricin 7-*O*-{3′-*O*-coumaroyl-[glucuronopyranosyl-(1-2′)]-*O*-glucopyranosyl-(1-2)-*O*-glucuronopyranoside}	C_44_H_46_O_26_	990.2277	991, 829, 507, 331	989, 659, 495
16	Tricin 7-*O*-glucuronopyranoside	C_23_H_22_O_13_	506.105	507, 331	505, 490, 329, 314
17	Tricin 7-*O*-[2′-*O*-sinapoyl-glucuronopyranosyl-(1-2)-*O*-glucuronopyranoside]	C_40_H_40_O_23_	888.196	889, 507, 383, 331, 207	887, 557, 399, 223
18	Chrysoeriol 7-*O*-[2′-*O*-feruloyl-glucuronopyranosyl-(1-2)-*O*-glucuronopyranoside]	C_38_H_36_O_21_	828.1749	829, 477, 353, 301, 177	827, 527, 333, 193
19	Tricin 7-*O*-[2′-*O*-feruloyl-glucuronopyranosyl-(1-2)-*O*-glucuronopyranoside]	C_39_H_38_O_22_	858.1854	859, 507, 331	857, 527, 193
20	Tricin 7-*O*-[2′-*O*-coumaroyl-glucuronopyranosyl-(1-2)-*O*-glucuronopyranoside]	C_38_H_36_O_21_	828.1749	829, 507, 331, 147	827, 497, 333, 163
21	Tricin 7-*O*-{3-*O*-feruloyl-[glucopyranosyl-(1-2′)-*O*-glucuronopyranosyl]-(1-2)-*O*-glucuronopyranoside}	C_45_H_48_O_27_	1020.238	1021, 683, 507, 339, 177	1019, 689, 495
22	Tricin 5-*O*-glucuronopyranosyl-7-*O*-glucopyranoside	C_29_H_32_O_18_	668.1588	669, 507, 331	667, 491, 329
23	Tricin 7-*O*-[2′-*O*-5-hydroxyferuloyl-glucuronopyranosyl-(1-2)-*O*-glucuronopyranoside]	C_39_H_38_O_23_	874.1803	875, 507, 369, 331	873, 543, 351, 209
24	Chrysoeriol 4′-*O*-xylopyranosyl-7-*O*-[2′-*O*-coumaroyl-glucuronopyranosyl-(1-2)-*O*-glucuronopyranoside]	C_42_H_42_O_24_	930.2066	931, 799, 609, 433, 301	929, 797, 497, 163
25	Tricin 4′-*O*-xylopyranosyl-7-*O*-[2′-*O*-coumaroyl-glucuronopyranosyl-(1-2)-*O*-glucuronopyranoside]	C_43_H_44_O_25_	960.2171	961, 829, 507, 463, 331	959, 827, 497
26	Tricin 7-*O*-[glucuronopyranosyl-(1-2)-*O*-methyloglucuronopyranoside]	C_30_H_32_O_19_	696.1537	697, 521, 331	695, 533, 329
27	Isorhamnetin 3(7)-*O*-[2′-*O*-coumaroyl-glucuronopyranosyl-(1-2)-*O*-glucuronopyranoside]	C_37_H_34_O_21_	814.1592	815, 493, 317	813, 649, 497, 315, 163
28	Chrysoeriol 7-O-glucuronopyranoside	C_22_H_20_O_12_	476.0954	477, 301	-
29	Apigenin 7-*O*-[2′-*O*-feruloyl-glucuronopyranosyl-(1-2)-*O*-glucuronopyranoside]	C_37_H_34_O_20_	798.1643	799, 447, 331, 271	797, 603, 527, 333, 193
30	Chrysoeriol 7-*O*-[2′-*O*-coumaroyl-glucuronopyranosyl-(1-2)-*O*-glucuronopyranoside]	C_37_H_34_O_20_	798.1643	799, 477, 301	797, 497
31	Tricin 7-*O*-[2′-*O*-feruloyl-glucuronopyranosyl-(1-2)-*O*-methyloglucuronopyranoside]	C_40_H_40_O_22_	872.2011	873, 521, 331, 177	871, 677, 519, 329
32	Tricin 7-*O*-[2′-*O*-benzoyl-glucuronopyranosyl-(1-2)-*O*-glucuronopyranoside]	C_36_H_34_O_20_	786.1643	787, 507, 331	785, 455, 333, 121
33	Tricin 7-*O*-[2′-*O*-coumaroyl-methyloglucuronopyranosyl-(1-2)-*O*-glucuronopyranoside]	C_39_H_38_O_21_	842.1905	843, 507, 331	841, 695, 633, 511, 329, 163
34	Tricin 7-*O*-[2′-*O*-dehydrodiferuloyl-glucopyranosyl-(1-2)-*O*-glucuronopyranoside]	C_49_H_48_O_25_	1036.2479	1037, 531, 331	1035, 705, 547, 371
35	Tricin 7-*O*-[2′-*O*-dehydrodiferuloyl-glucuronopyranosyl-(1-2)-*O*-glucuronopyranoside]	C_49_H_48_O_26_	1052.2428	1053, 859, 507, 331	1051, 721, 563, 387

## Supplementary Materials

Supplementary materials can be accessed at: http://www.mdpi.com/1420-3049/21/9/1229/s1.

## References

[1] Smolarz H.D., Budzianowski J., Bogucka-Kocka A., Kocki J., Mendyk E. (2008). Flavonoid glucuronides with anti-leukaemic activity from *Polygonum amphibium* L.. Phytochem. Anal..

[2] Poe M.L., Bates A., Onyilagha J. (2013). Distribution of Leaf Flavonoid Aglycones and Glucuronides in the Genus Phaseolus and Related Genera. Int. J. Biol..

[3] Manach C., Scalbert A., Morand C., Rémésy C., Jiménez L. (2004). Polyphenols: Food sources and bioavailability. Am. J. Clin. Nutr..

[4] Andersen O.M., Markham K.R. (2006). Flavonoids. Chemistry, Biochemistry and Applications.

[5] Nazaruk J., Gudej J. (2003). Flavonoid compounds from the flowers of *Cirsium rivulare* (Jacq.) All. Acta Pol. Pharm. Drug Res..

[6] Zhang Z., Liu Y., Luo P., Zhang H. (2009). Separation and Purification of Two Flavone Glucuronides from *Erigeron multiradiatus* (Lindl.) Benth with Macroporous Resins. J. Biomed. Biotechnol..

[7] Williams C.A., Harborne J.B., Geiger H., Robin J., Hoult S. (1999). The flavonoids of *Tanacetum parthenium* and T-vulgare and their anti-inflammatory properties. Phytochemistry.

[8] Granica S., Czerwińska M.E., Zyzyńska-Granica B., Kiss A.K. (2013). Antioxidant and anti-inflammatory flavonol glucuronides from *Polygonum aviculare* L.. Fitoterapia.

[9] Tomimori T., Miyaichi Y., Imoto Y., Kizu H., Suzuki C. (1984). Studies on the constituents of Scutellaria species. IV. On the flavonoid constituents of the root of *Scutellaria baicalensis* Georgi (4). Yakugaku Zasshi.

[10] Liu G., Rajesh N., Wang X., Zhang M., Wu Q., Li S., Chen B., Yao S. (2011). Identification of flavonoids in the stems and leaves of *Scutellaria baicalensis* Georgi. J. Chromatogr. B.

[11] Lu Y., Yeap Foo L. (2000). Flavonoid and phenolic glycosides from *Salvia officinalis*. Phytochemistry.

[12] Al-Qudah M.A., Al-Jaber H.I., Abu Zarga M.H., Abu Orabi S.T. (2014). Flavonoid and phenolic compounds from *Salvia palaestina* L. growing wild in Jordan and their antioxidant activities. Phytochemistry.

[13] Huang Y., De Bruyne T., Apers S., Ma Y., Claeys M., Pieters L., Vlietinck A. (1999). Flavonoid glucuronides from *Picria fel-terrae*. Phytochemistry.

[14] Yamazaki K., Iwashina T., Kitajima J., Gamou Y., Yoshida A., Tannowa T. (2007). External and internal flavonoids from Madagascarian *Uncarina* species (Pedaliaceae). Biochem. Syst. Ecol..

[15] Aritomi M., Kawasaki T. (1984). Three highly oxygenated flavone glucuronides in leaves of *Spinacia oleracea*. Phytochemistry.

[16] Kowalska I., Stochmal A., Kapusta I., Janda B., Pizza C., Piacente S., Oleszek W. (2007). Flavonoids from barrel medic (*Medicago truncatula*) aerial parts. J. Agric. Food Chem..

[17] Stochmal A., Piacente S., Pizza C., De Riccardis F., Leitz R., Oleszek W. (2001). Alfalfa (*Medicago sativa* L.) Flavonoids. 1. Apigenin and luteolin glycosides from aerial parts. J. Agric. Food Chem..

[18] Stochmal A., Simonet A.M., Macias F.A., Oleszek W. (2001). Alfalfa (*Medicago sativa* L.) flavonoids. 2. Tricin and chrysoeriol glycosides from aerial parts. J. Agric. Food Chem..

[19] Marczak L., Stobiecki M., Jasinski M., Oleszek W., Kachlicki P. (2010). Fragmentation pathways of acylated flavonoid diglucuronides from leaves of *Medicago truncatula*. Phytochem. Anal..

[20] Yoshida K., Kameda K., Kondot T. (1993). Diglucuronoflavones from purple leaves of *Perilla ocimoides*. Phytochemistry.

[21] Schulz M., Strack D., Weissenböck G., Markham K.R., Dellamonica G., Chopin J. (1985). Two luteolin *O*-glucuronides from primary leaves of *Secale cereale*. Phytochemistry.

[22] Mues R. (1983). Species Specific Flavone Glucuronides in Elodea Species. Biochem. Syst. Ecol..

[23] Pawlak K., Bylka W., Jazurek B., Matlawska I., Sikorska M., Manikowski H., Bialek-Bylka G. (2010). Antioxidant activity of flavonoids of different polarity, assayed by modified ABTS cation radical decolorization and EPR technique. Acta Biol. Crac. Ser. Bot..

[24] Budzianowski J., Korzeniowska K., Chmara E., Mrozikiewicz A. (1999). Microvascular protective activity of flavonoid glucuronides fraction from *Tulipa gesneriana*. Phytother. Res..

[25] Wu Z.Y., Raven P.H., Hong D.Y. (2003). Flora of China. Vol. 5 (Ulmaceae through Basellaceae).

[26] Aswal B.S., Bhakuni D.S., Goel A.K., Kar K., Mehrotra B.N. (1984). Screening of Indian plants for biological activity—Part XI. Indian J. Exp. Biol..

[27] Stobiecki M. (2001). Applications of separation techniques hyphenated to mass spectrometer for metabolic profiling. Curr. Org. Chem..

[28] March R.E., Miao X.S., Metcalfe C.D., Stobiecki M., Marczak L. (2004). A fragmentation study of an isoflavone glycoside, genistein-7-*O*-glucoside, using electrospray quadrupole time-of-flight mass spectrometry at high mass resolution. Int. J. Mass Spectrom..

[29] Shao Y., Jiang J., Ran L., Lu C., Wei C., Wang Y. (2014). Analysis of Flavonoids and Hydroxycinnamic Acid Derivatives in Rapeseeds (*Brassica napus* L. var. napus) by HPLC-PDA-ESI(−)-MSn/HRMS. J. Agric. Food Chem..

[30] Wojakowska A., Perkowski J., Góral T., Stobiecki M. (2013). Structural characterization of flavonoid glycosides from leaves of wheat (*Triticum aestivum* L.) using LC/MS/MS profiling of the target compounds. J. Mass Spectrom..

[31] Abad-García B., Garmón-Lobato S., Berrueta L.A., Gallo B., Vicente F. (2009). Practical guidelines for characterization of *O*-diglycosyl flavonoid isomers by triple quadrupole MS and their applications for identification of some fruit juices flavonoids. J. Mass Spectrom..

[32] Pikulski M., Brodbelt J.S. (2003). Differentiation of flavonoid glycoside isomers by using metal complexation and electrospray ionization mass spectrometry. J. Am. Soc. Mass Spectrom..

[33] Lv Y.-W., Hu W., Wang Y.-L., Huang L.-F., He Y.-B., Xie X.-Z. (2011). Identification and Determination of Flavonoids in Astragali Radix by High Performance Liquid Chromatography Coupled with DAD and ESI-MS Detection. Molecules.

[34] Feng M., Zhu Z., Zuo L., Chen L., Yuan Q., Shan G., Luo S.-Z. (2015). A strategy for rapid structural characterization of saponins and flavonoids from the testa of *Camellia oleifera* Abel seeds by ultra-high-pressure liquid chromatography combined with electrospray ionization linear ion trap-orbitrap mass spectrometry. Anal. Methods.

[35] Wang S., Chen L., Leng J., Chen P., Fan X., Cheng Y. (2014). Fragment ion diagnostic strategies for the comprehensive identification of chemical profile of Gui-Zhi-Tang by integrating high-resolution MS, multiple-stage MS and UV information. J. Pharm. Biomed. Anal..

[36] Sarker S.D., Sik V., Rees H.H., Dinan L. (1998). 1α,20*R*-dihydroxyecdysone from *Axyris amaranthoides*. Phytochemistry.

[37] Dinan L. (1995). Distribution of phytoecdysteroids within Chenopodiaceae. Eur. J. Entomol..

[38] Wojakowska A., Piasecka A., García-López P.M., Zamora-Natera F., Krajewski P., Marczak Ł., Kachlicki P., Stobiecki M. (2013). Structural analysis and profiling of phenolic secondary metabolites of Mexican lupine species using LC-MS techniques. Phytochemistry.

[39] Antunes-Ricardo M., Moreno-García B.E., Gutiérrez-Uribe J.A., Aráiz-Hernández D., Alvarez M.M., Serna-Saldivar S.O. (2014). Induction of Apoptosis in Colon Cancer Cells Treated with Isorhamnetin Glycosides from Opuntia Ficus-indica Pads. Plant Foods Hum. Nutr..

[40] Sikorska M., Matlawska I. (2001). Kaempferol, isorhamnetin and their glycosides in the flowers of *Asclepias syriaca* L.. Acta Pol. Pharm. Drug Res..

[41] Domon B., Costello C.E. (1988). A systematic nomenclature for carbohydrate fragmentations in FAB-MS/MS spectra of glycoconjugates. Glycoconj. J..

[42] Vukics V., Guttman A. (2008). Structural characterization of flavonoid glycosides by multi-stage mass spectrometry. Mass Spectrom. Rev..

[43] Kachlicki P., Einhorn J., Muth D., Kerhoas L., Stobiecki M. (2008). Evaluation of glycosylation and malonylation patterns in flavonoid glycosides during LC/MS/MS metabolite profiling. J. Mass Spectrom..

[44] Lee W.J., Choi S.W. (2012). Quantitative changes of polyphenolic compounds in mulberry (Morus alba L.) leaves in relation to varieties, harvest period, and heat processing. Prev. Nutr. Food Sci..

[45] Mullen W., Hartley R.C., Crozier A. (2003). Detection and identification of ^14^C-labelled flavonol metabolites by high-performance liquid chromatography-radiocounting and tandem mass spectrometry. J. Chromatogr. A.

[46] Dobberstein D., Bunzel M. (2010). Identification of ferulate oligomers from corn stover. J. Sci. Food Agric..

[47] Renger A., Steinhart H. (2000). Ferulic acid dehydrodimers as structural elements in cereal dietary fibre. Eur. Food Res. Technol..

[48] Ford W.C., Hartley D.R. (1989). GC/MS characterization of cyclodimers from *p*-coumaric and ferulic acids by photodimerization—A possible factor influencing cell wall biodegradability. J. Sci. Food Agric..

[49] Micard V., Grabber J.H., Ralph J., Renard C.M.G.C., Thibault J.F. (1997). Dehydrodiferulic acids from sugar-beet pulp. Phytochemistry.

[50] Stobiecki M., Busko M., Marczak L., Bednarek P., Pislewska M., Wojtaszek P. (2002). The complexity of oxidative cross-linking of phenylpropanoids: Evidence from an in vitro model system. Funct. Plant Biol..

[51] Svedström U., Vuorela H., Kostiainen R., Laakso I., Hiltunen R. (2006). Fractionation of polyphenols in hawthorn into polymeric procyanidins, phenolic acids and flavonoids prior to high-performance liquid chromatographic analysis. J. Chromatogr. A.

