# Supplementary material for: The Use of Mass Spectrometric Techniques to Differentiate Isobaric and Isomeric Flavonoid Conjugates from Axyris amaranthoides
Source: Molecules. 2016 Sep 19;21(9):1229. doi: 10.3390/molecules21091229 (PMC6273421; doi:10.3390/molecules21091229)
Supplement: Supplementary file 1 [file molecules-21-01229-s001.pdf]

Supplementary Materials: The Use of Mass Spectrometric Techniques to Differentiate Isobaric and Isomeric Flavonoid Conjugates from *Axyris amaranthoides*

Łukasz Marczak, Paulina Znajdek-Awiżeń and Wiesława Bylka

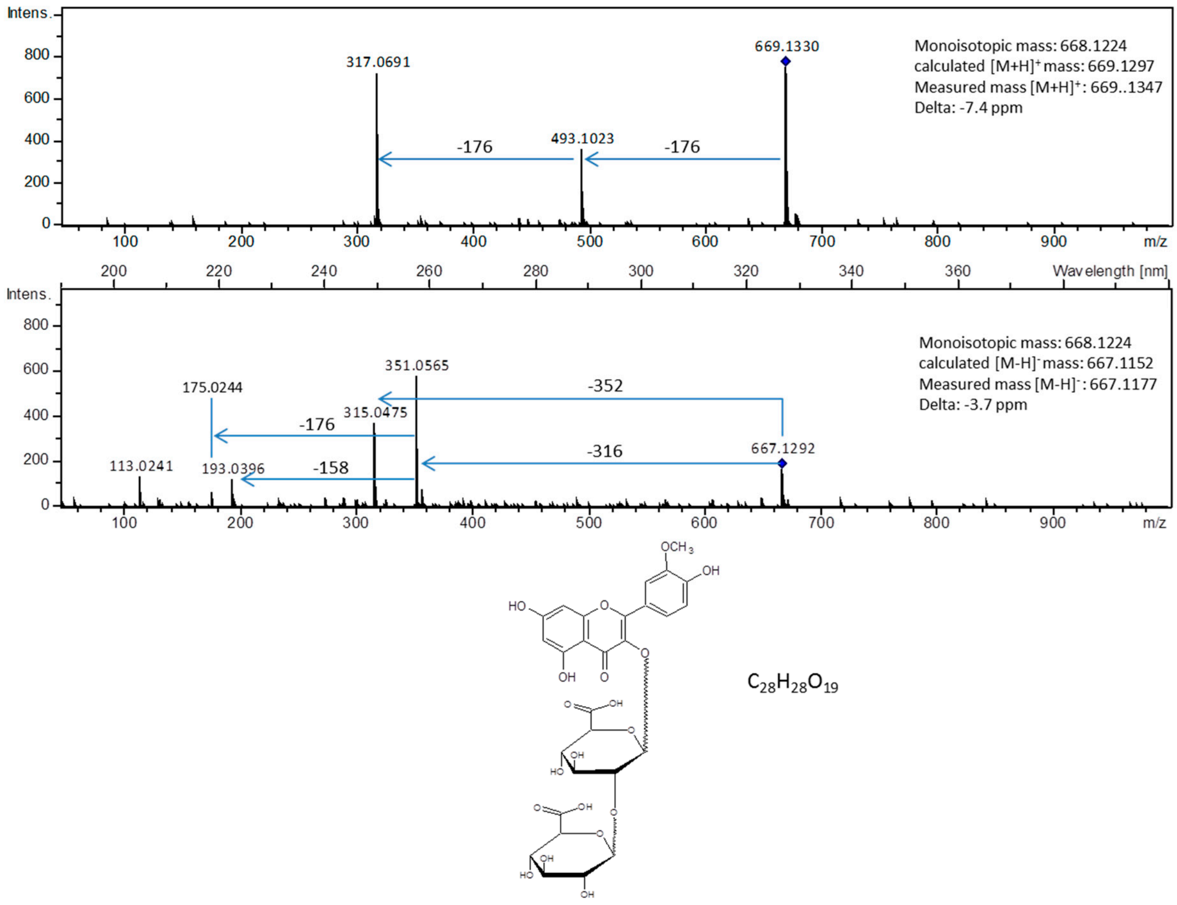

Figure S1. Isorhamnetin 7-*O*-glucuronopyranosyl-(1-2)-*O*-glucuronopyranoside.

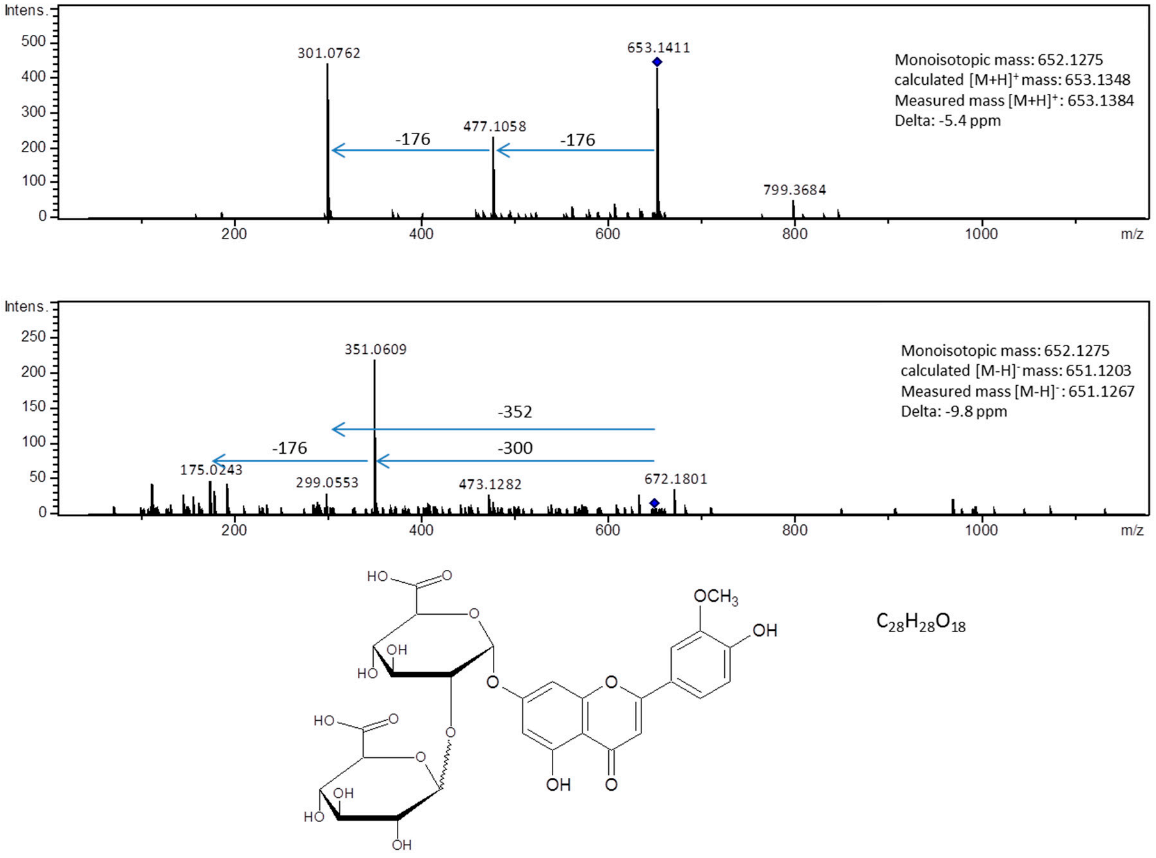

Figure S2. Chrysoeriol 7-*O*-glucuronopyranosyl-(1-2)-*O*-glucuronopyranoside.

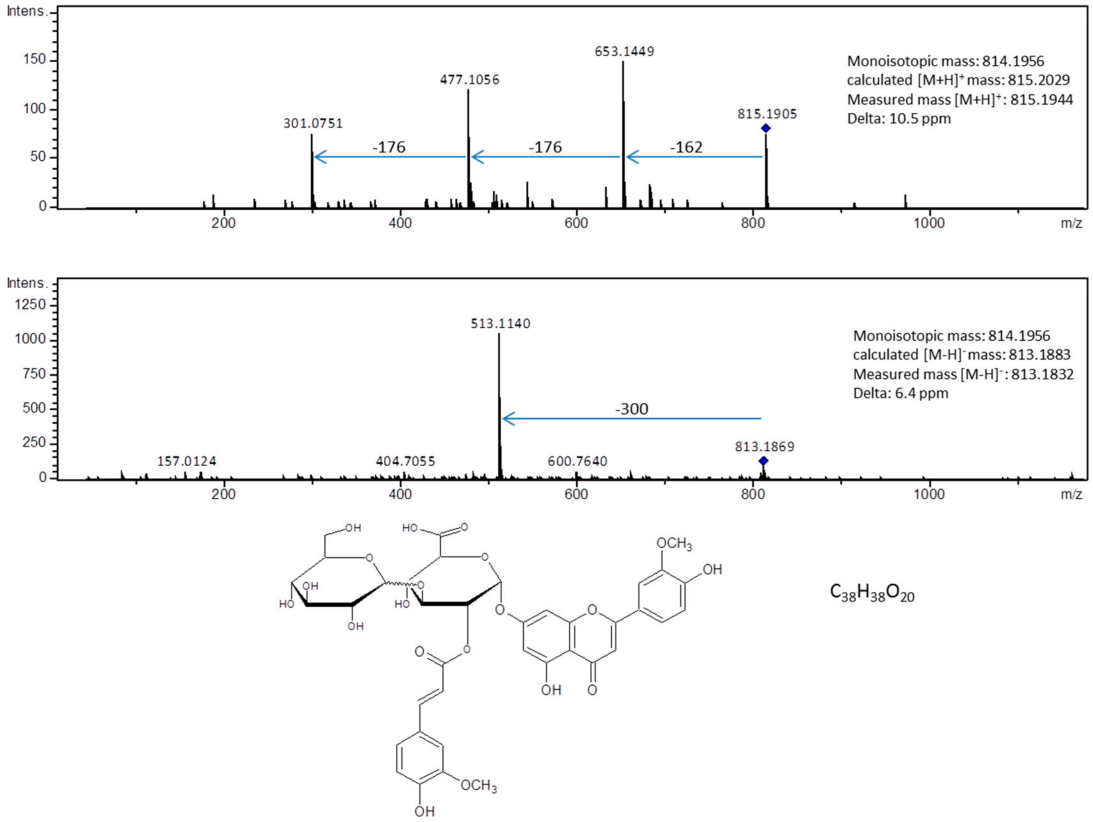

Figure S3. Chrysoeriol 7-*O*-[2-*O*-feruloyl-glucuronopyranosyl-(1-3)-*O*-glucopyranoside].

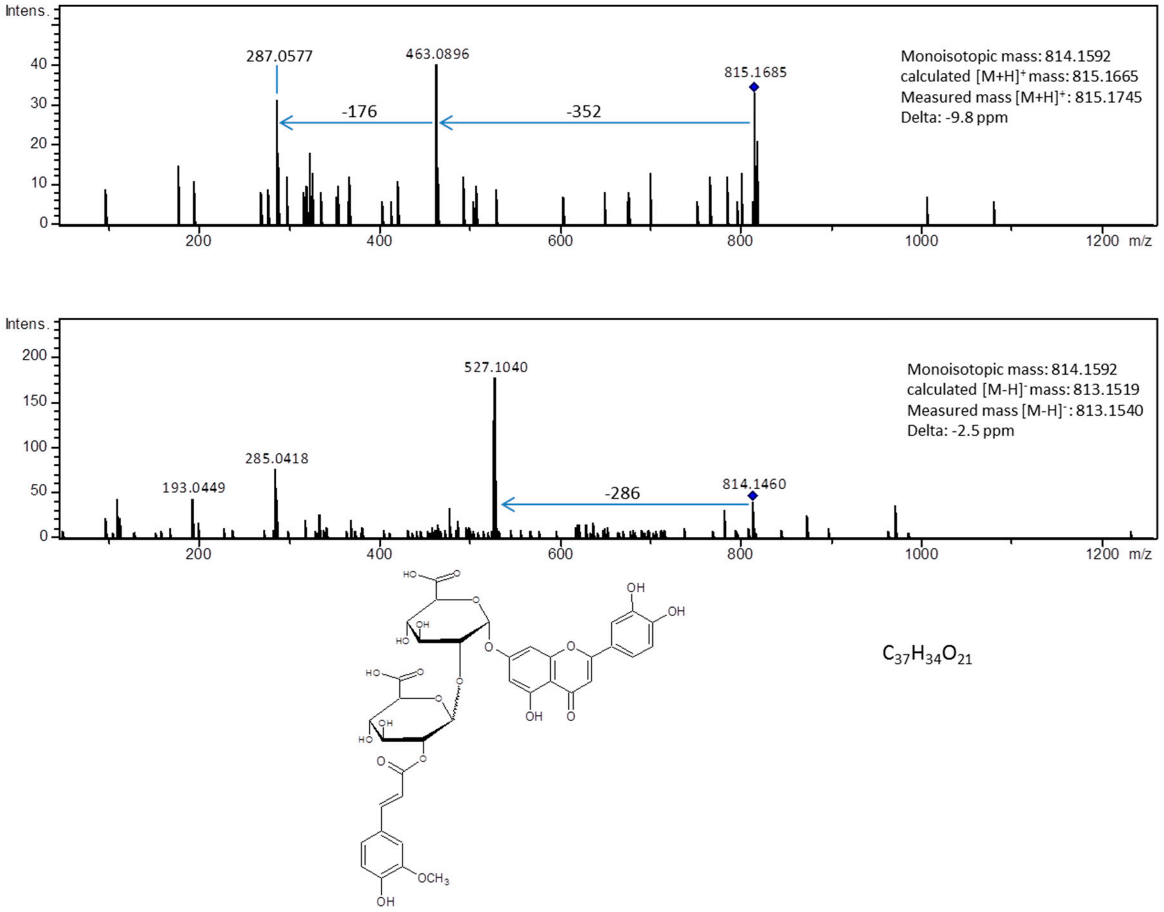

Figure S4. Luteolin 7-*O*-[2′-*O*-feruloyl-glucuronopyranosyl-(1-2)-*O*-glucuronopyranoside].

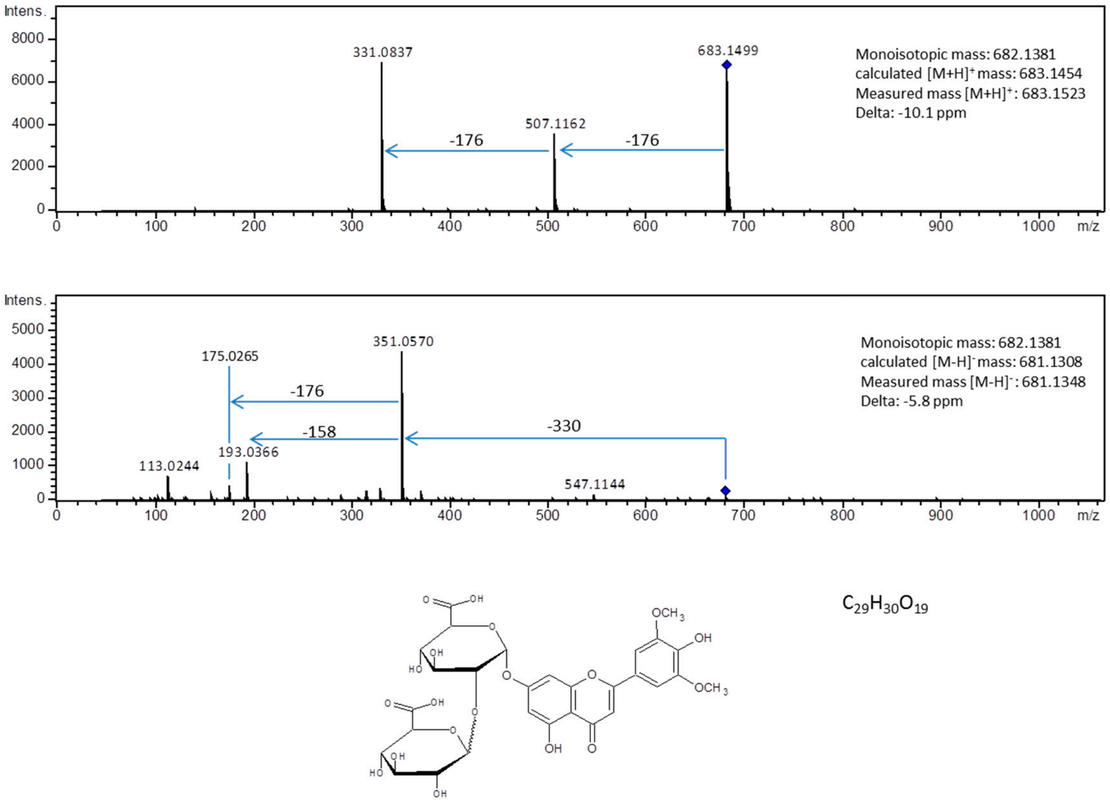

Figure S5. Tricin 7-*O*-glucuronopyranosyl-(1-2)-*O*-glucuronopyranoside.

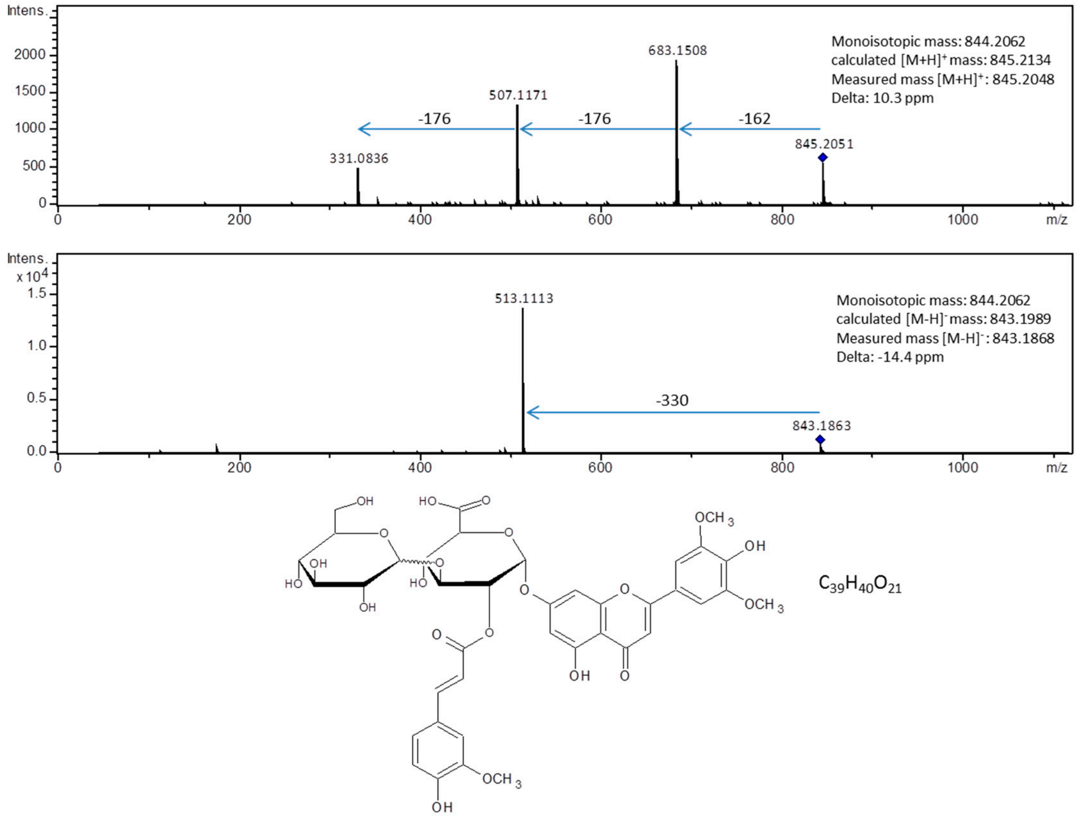

Figure S6. Tricin 7-*O*-[2-*O*-feruloyl-glucopyranosyl-(1-3)-*O*-glucuronopyranoside].

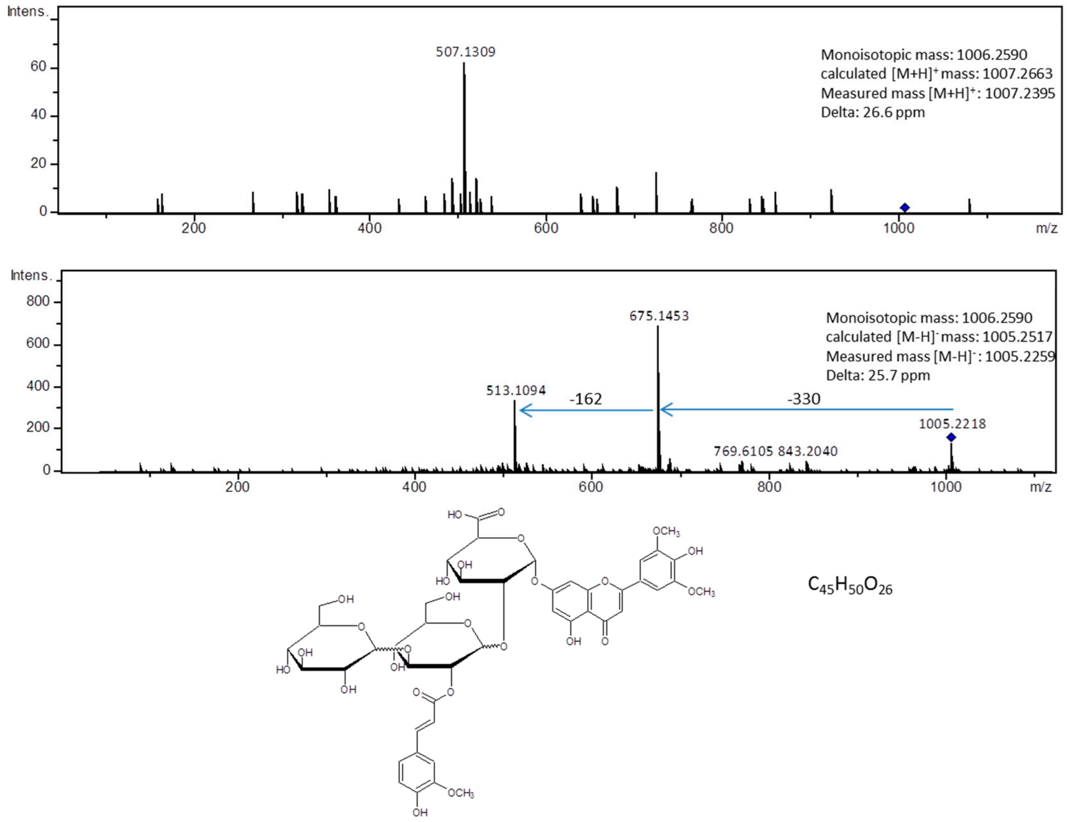

Figure S7. Tricin 7-*O*-{2′-*O*-feruloyl-[glucopyranosyl-(1-3′)-*O*-glucopyranosyl]-(1-2)-*O*-glucuronopyranoside}.

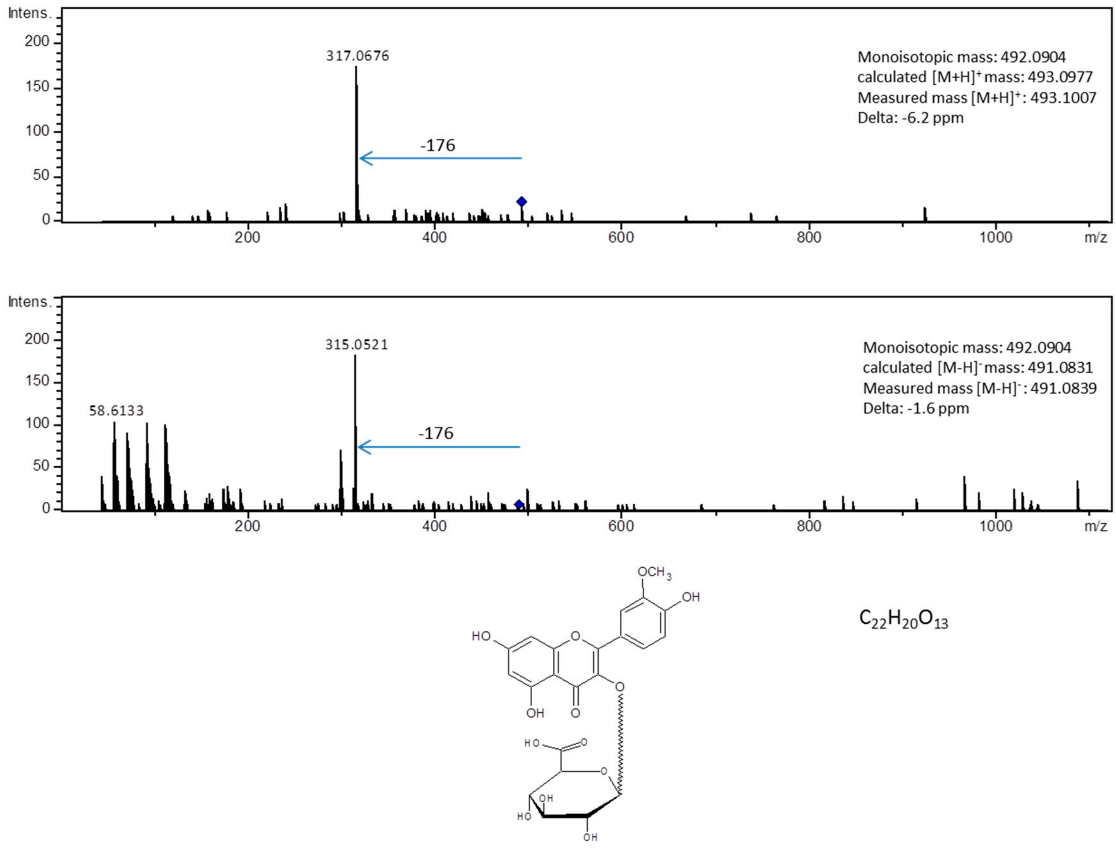

Figure S8. Isorhamnetin 3-*O*-glucuronopyranoside.

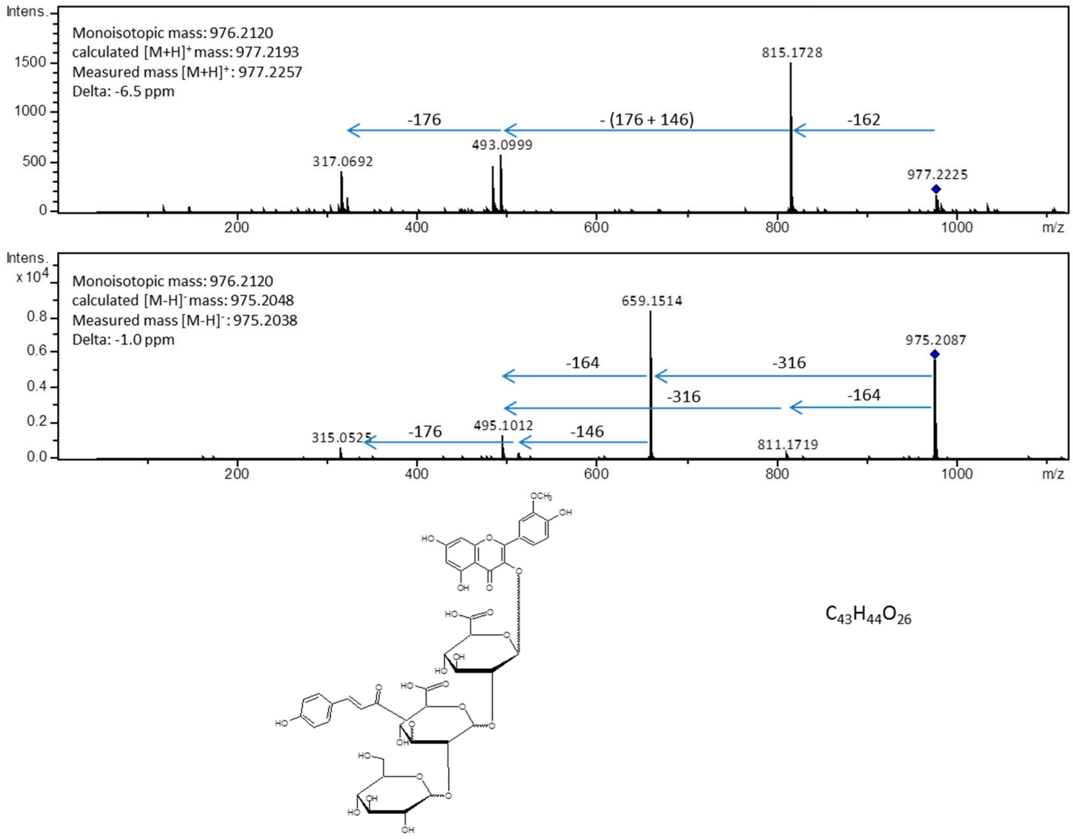

Figure S9. Isorhamnetin 3-*O*-{3′-*O*-coumaroyl-[glucuronopyranosyl-(1-2′)-*O*-glucopyranosyl]-(1-2)-*O*-glucuronopyranoside}.

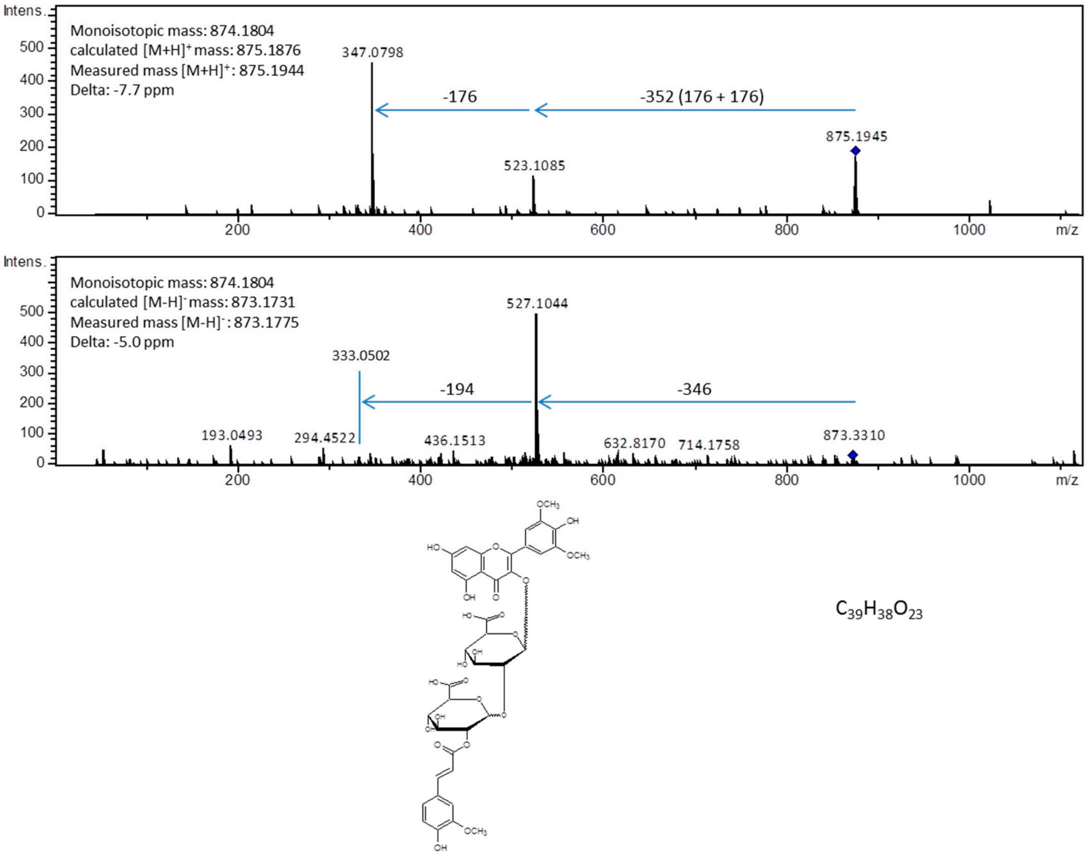

Figure S10. Syringetin 3-*O*-[2′-*O*-feuroyl-glucuronopyranosyl-(1-2)-*O*-glucuronopyranoside].

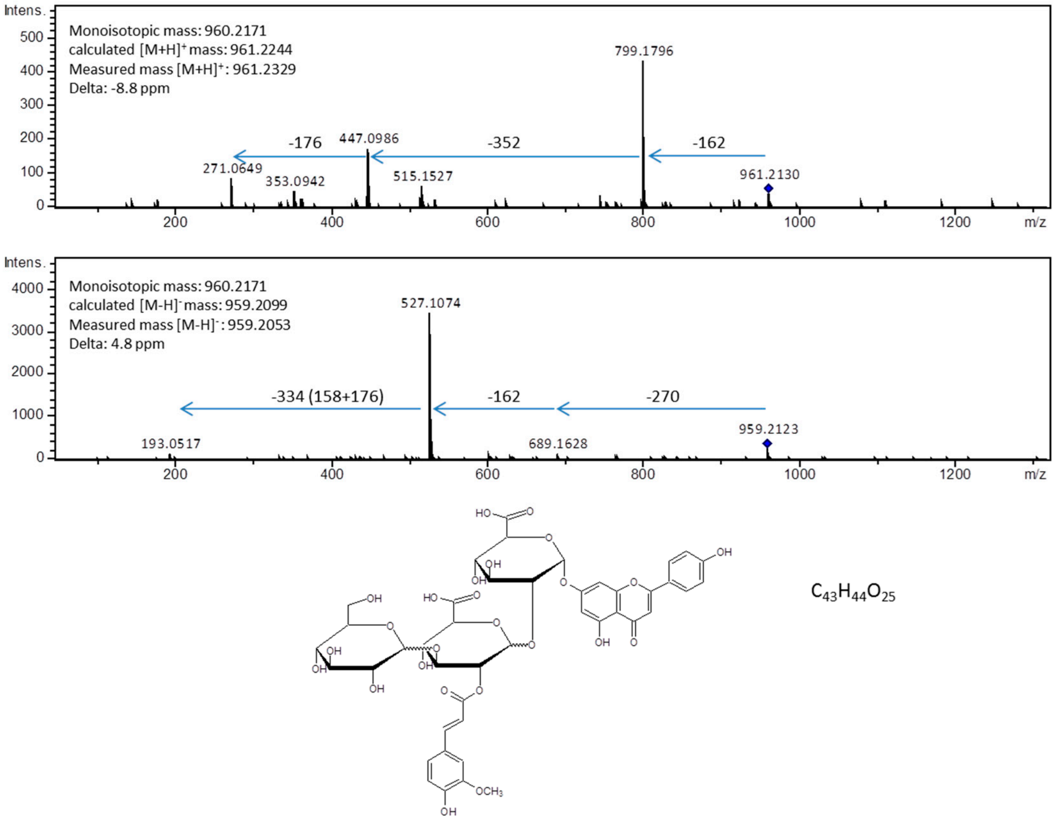

Figure S11. Apigenin 7-*O*-{2′-*O*-feruloyl-[glucopyranosyl-(1-3′)]-*O*-glucuronopyranosyl-(1-2)-*O*-glucuronopyranoside}.

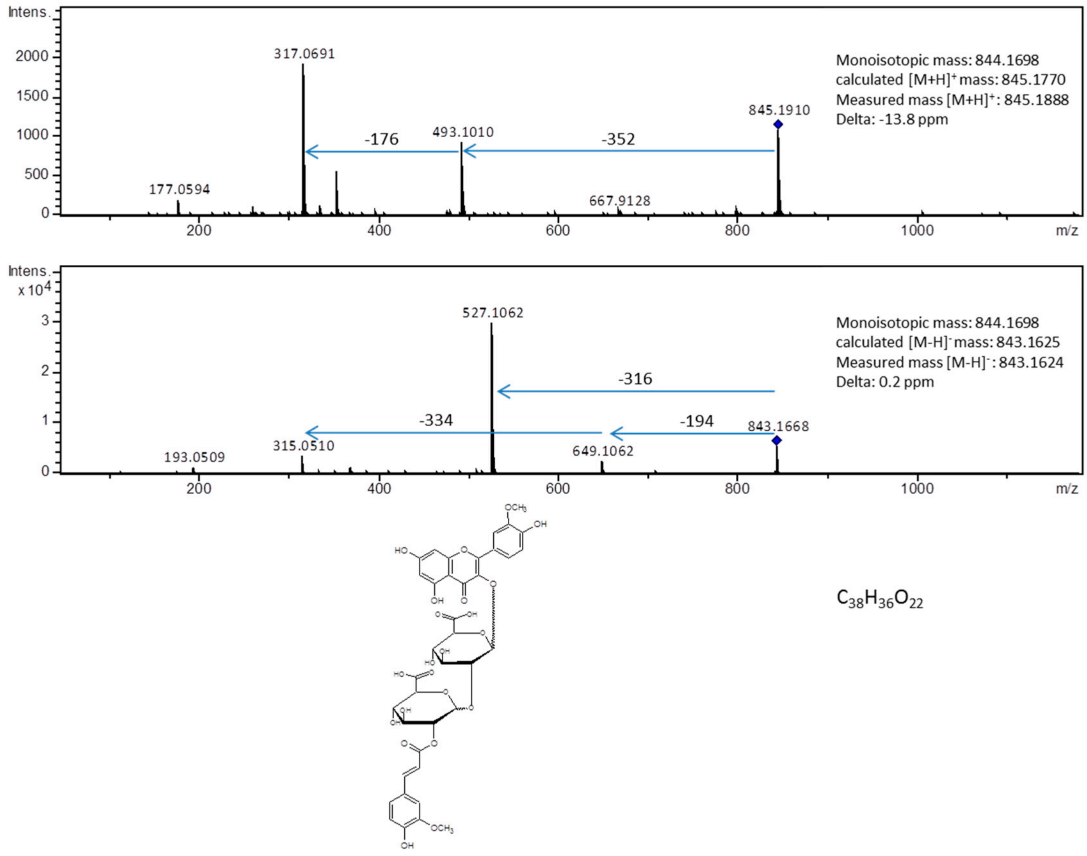

Figure S12. Isorhamnetin 3-*O*-[2′-*O*-feruloyl-glucuronopyranosyl-(1-2)-*O*-glucuronopyranoside].

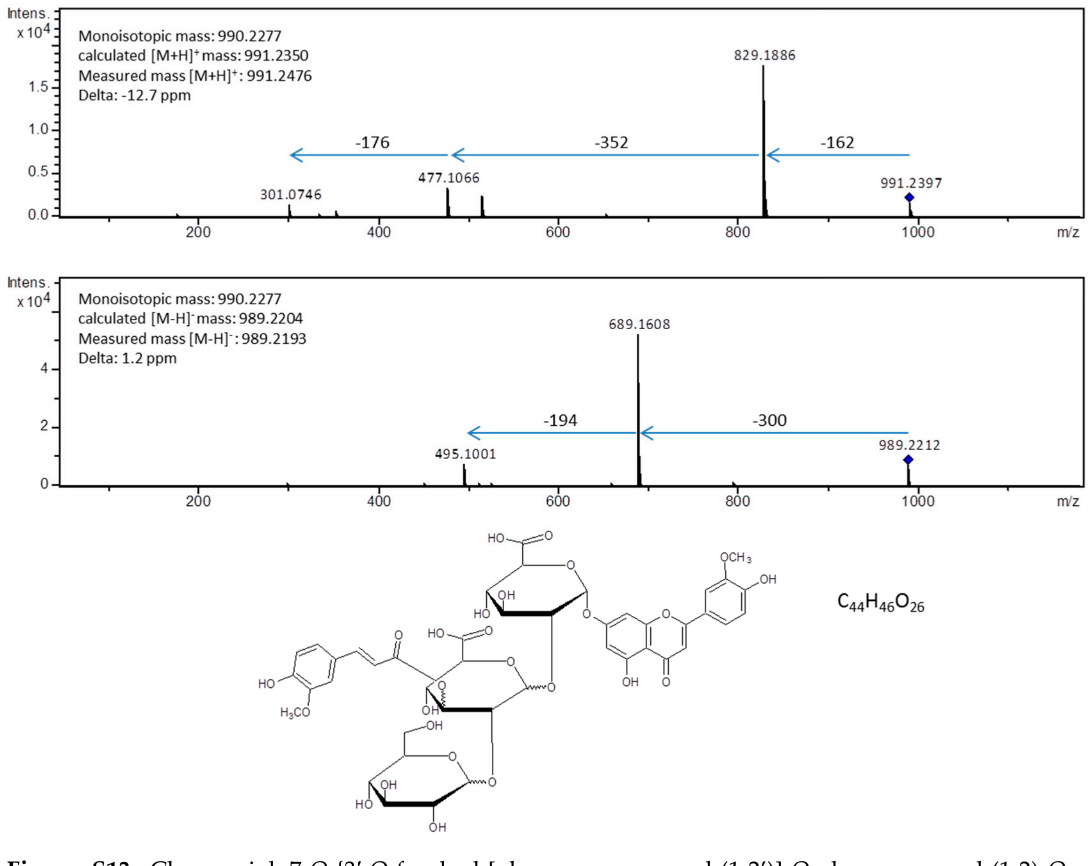

Figure S13. Chrysoeriol 7-*O*-{3′-*O*-feruloyl-[glucuronopyranosyl-(1-2′)]-*O*-glucopyranosyl-(1-2)-*O*-glucuronopyranoside}.

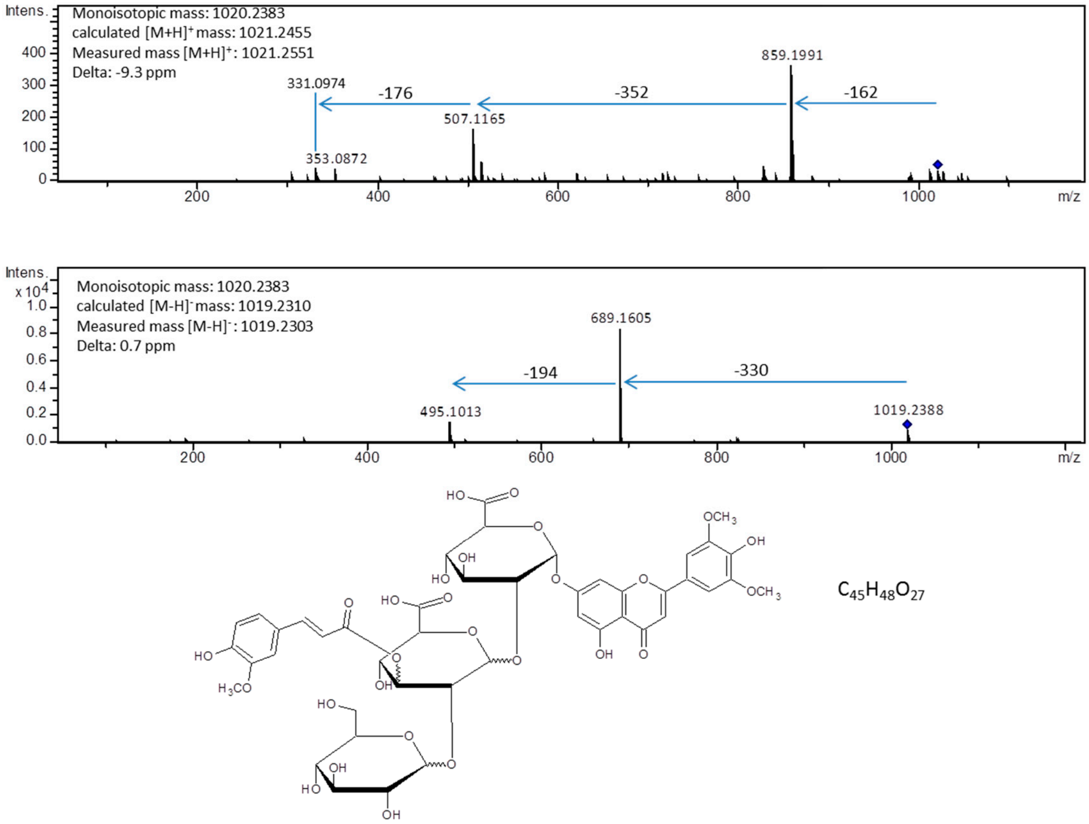

Figure S14. Tricin 7-*O*-{3′-*O*-feruloyl-[glucuronopyranosyl-(1-2)]-*O*-glucopyranosyl-(1-2)-*O*-glucuronopyranoside}.

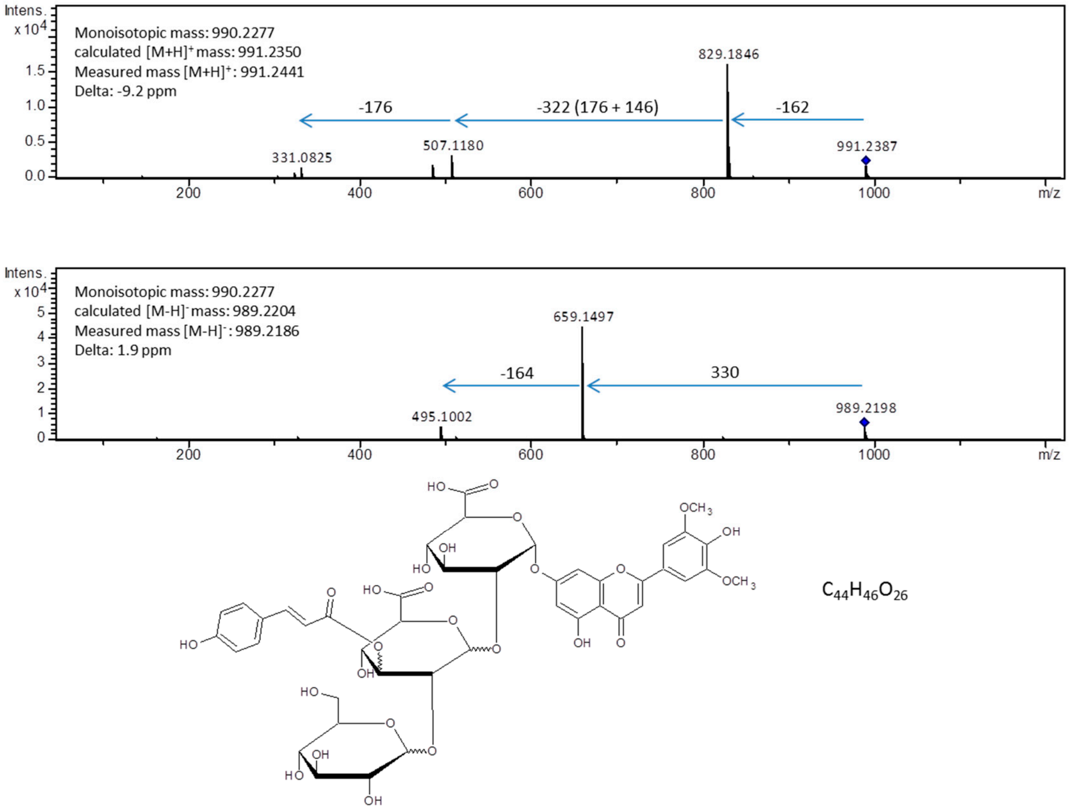

Figure S15. Tricin 7-*O*-{3′-*O*-coumaroyl-[glucuronopyranosyl-(1-2′)]-*O*-glucopyranosyl-(1-2)-*O*-glucuronopyranoside}.

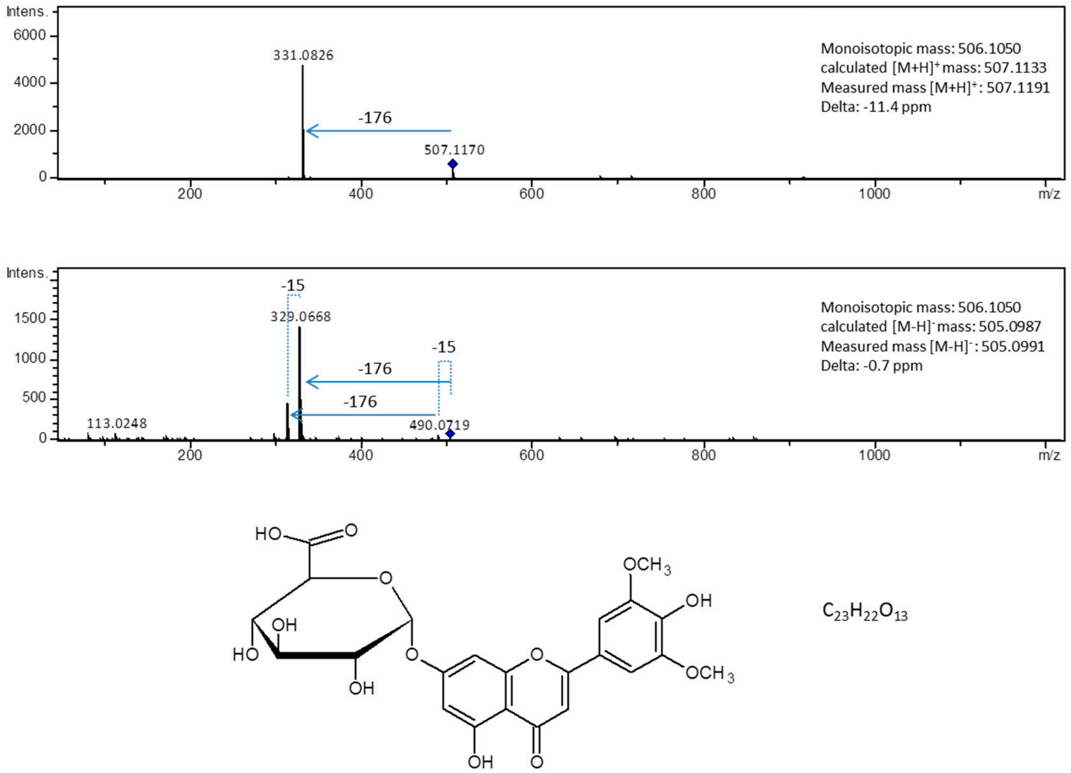

Figure S16. Tricin 7-*O*-glucuronopyranoside.

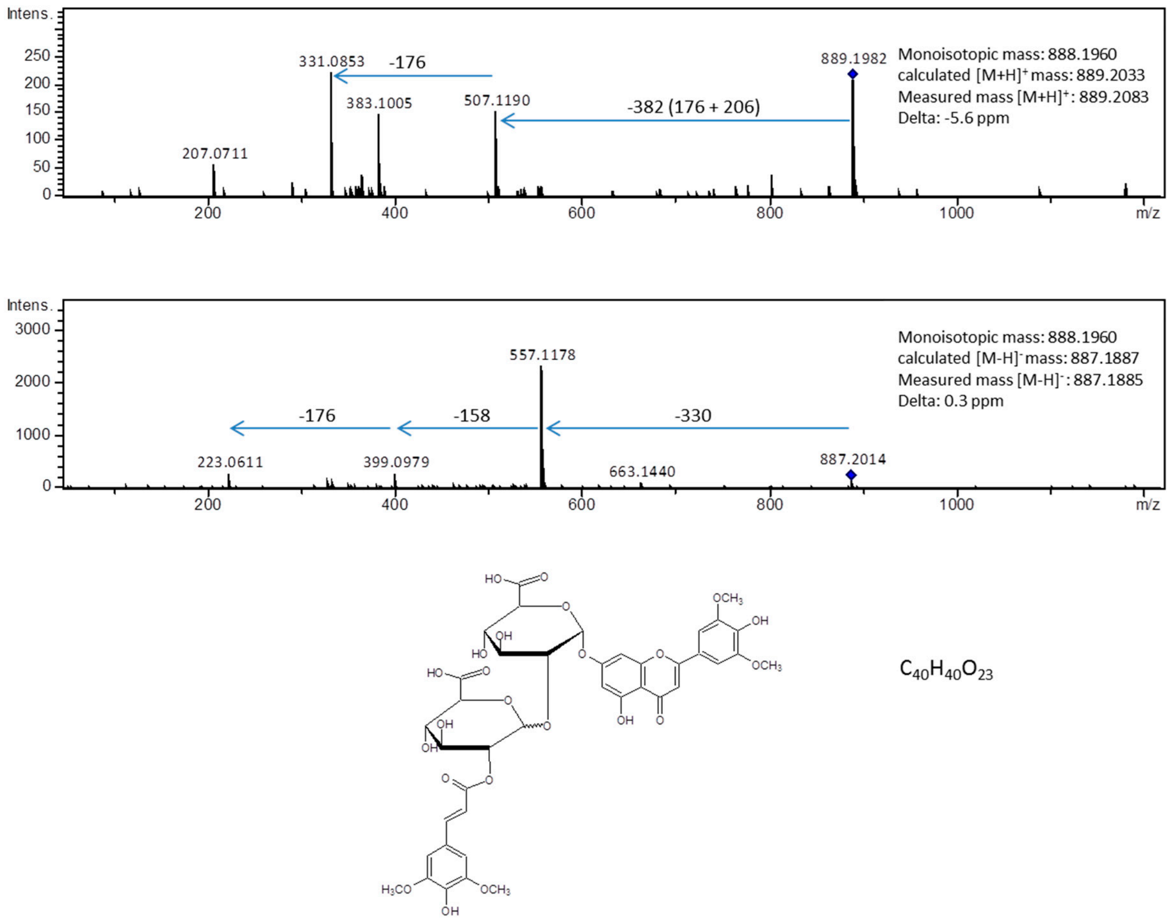

Figure S17. Tricin 7-*O*-[2′-*O*-sinapoyl-glucuronopyranosyl-(1-2)-*O*-glucuronopyranoside].

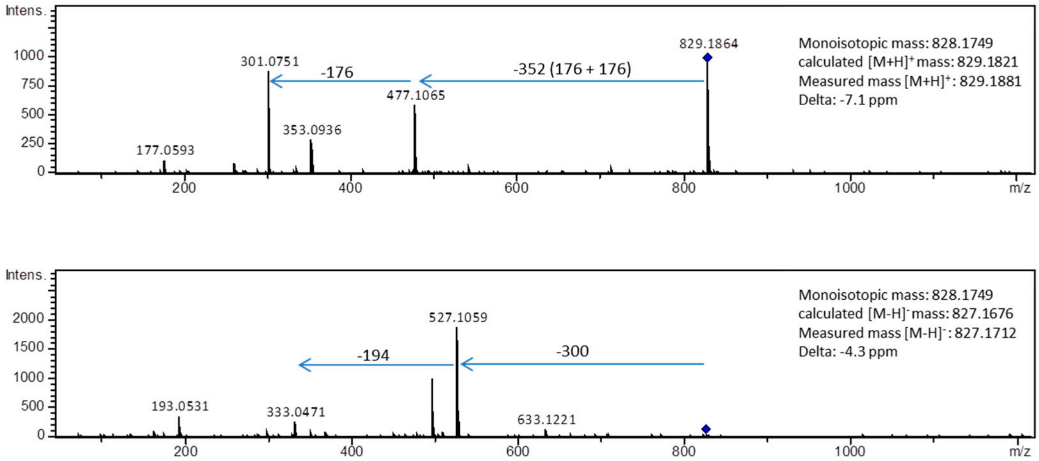

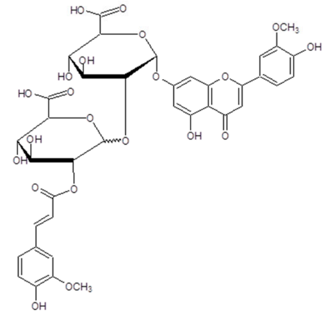

$C_{38}H_{36}O_{21}$

Figure S18. Chrysoeriol 7-*O*-[2′-*O*-feruloyl-glucuronopyranosyl-(1-2)-*O*-glucuronopyranoside].

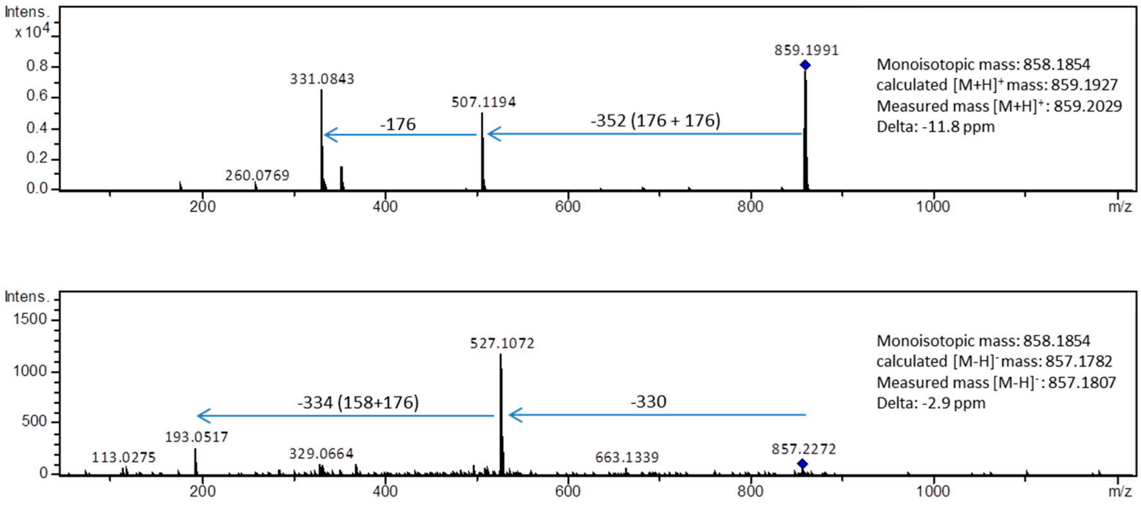

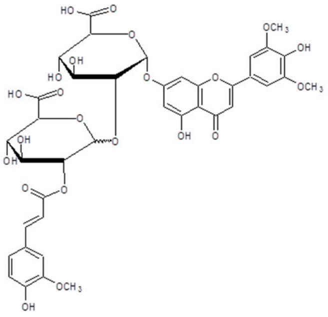

$C_{39}H_{38}O_{22}$

Figure S19. Tricin 7-*O*-[2′-*O*-feruloyl-glucuronopyranosyl-(1-2)-*O*-glucuronopyranoside].

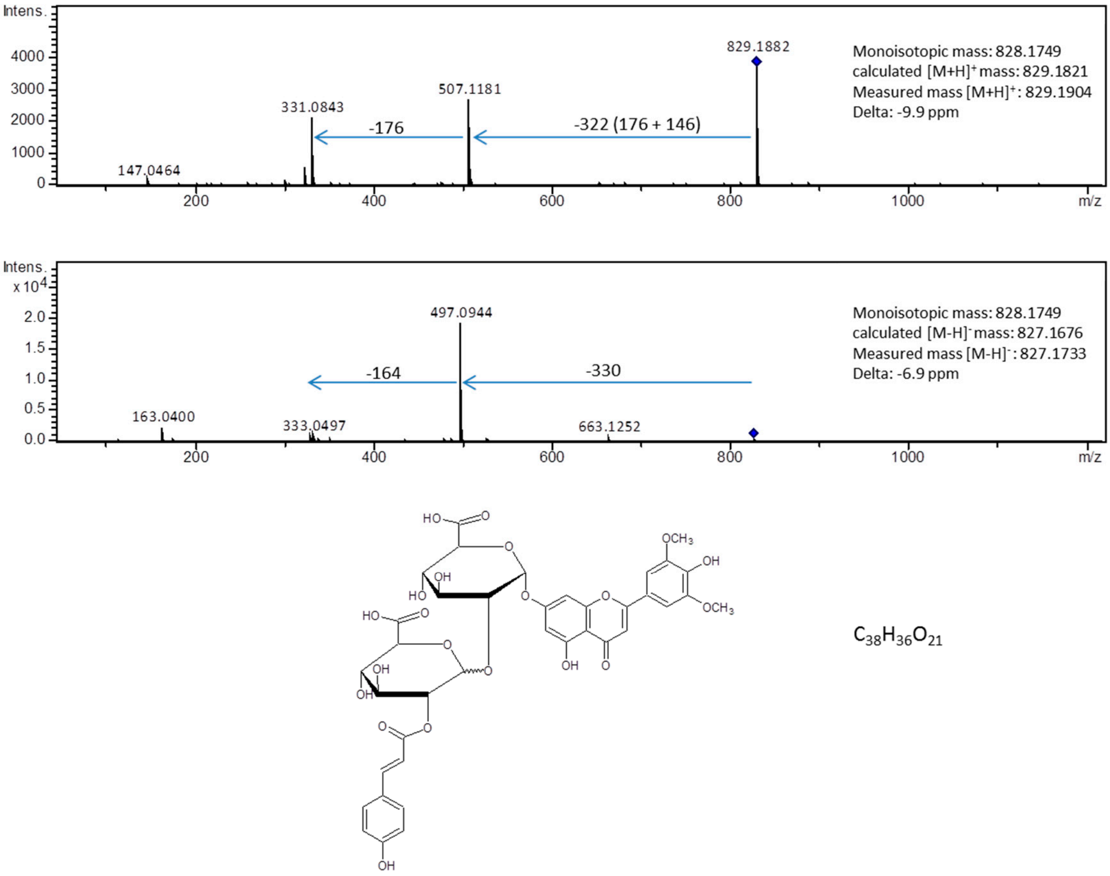

Figure S20. Tricin 7-*O*-[2′-*O*-coumaroyl-glucuronopyranosyl-(1-2)-*O*-glucuronopyranoside].

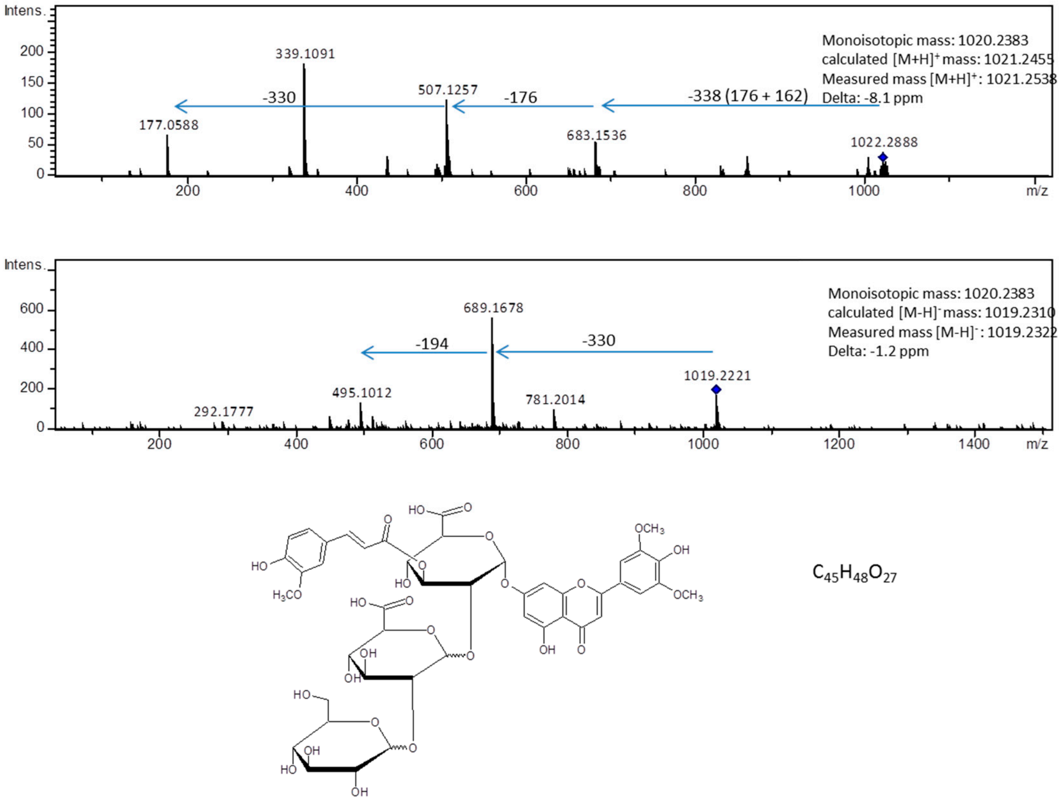

Figure S21. Tricin 7-*O*-{3-*O*-feruloyl-[glucopyranosyl-(1-2′)-*O*-glucuronopyranosyl]-(1-2)-*O*-glucuronopyranoside}.

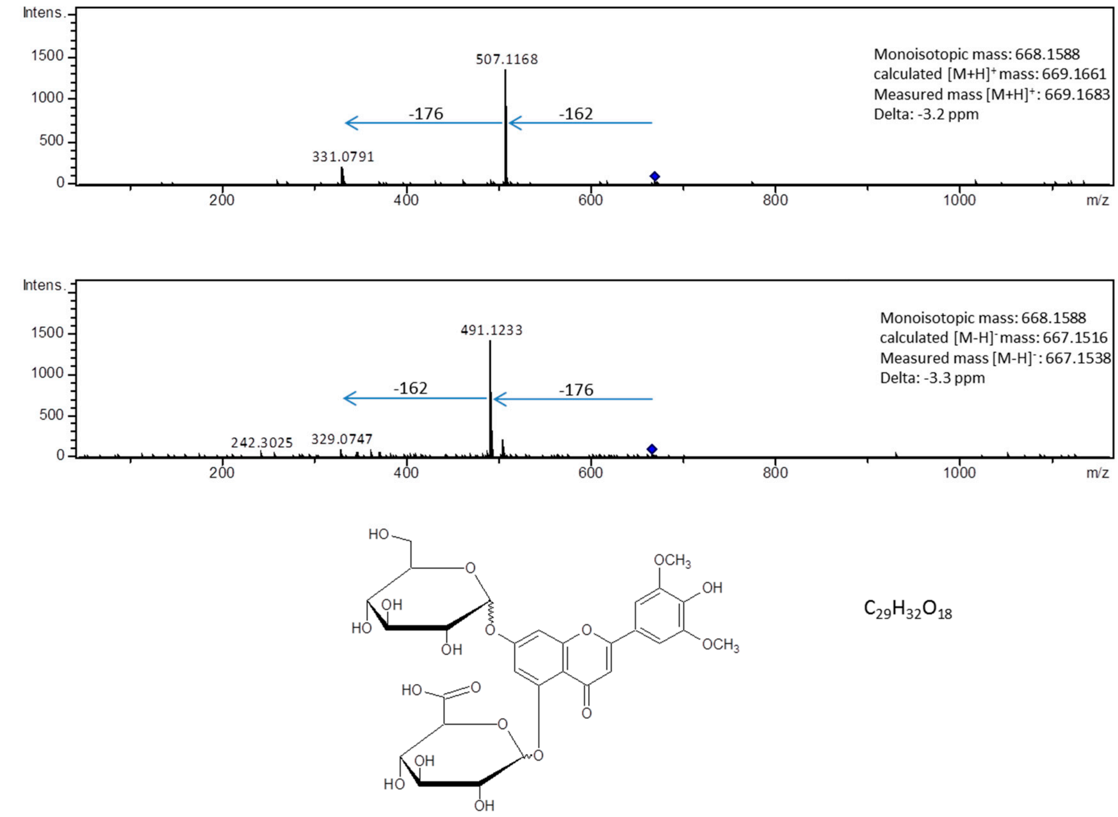

Figure S22. Tricin 5-*O*-glucuronopyranosyl-7-*O*-glucopyranoside.

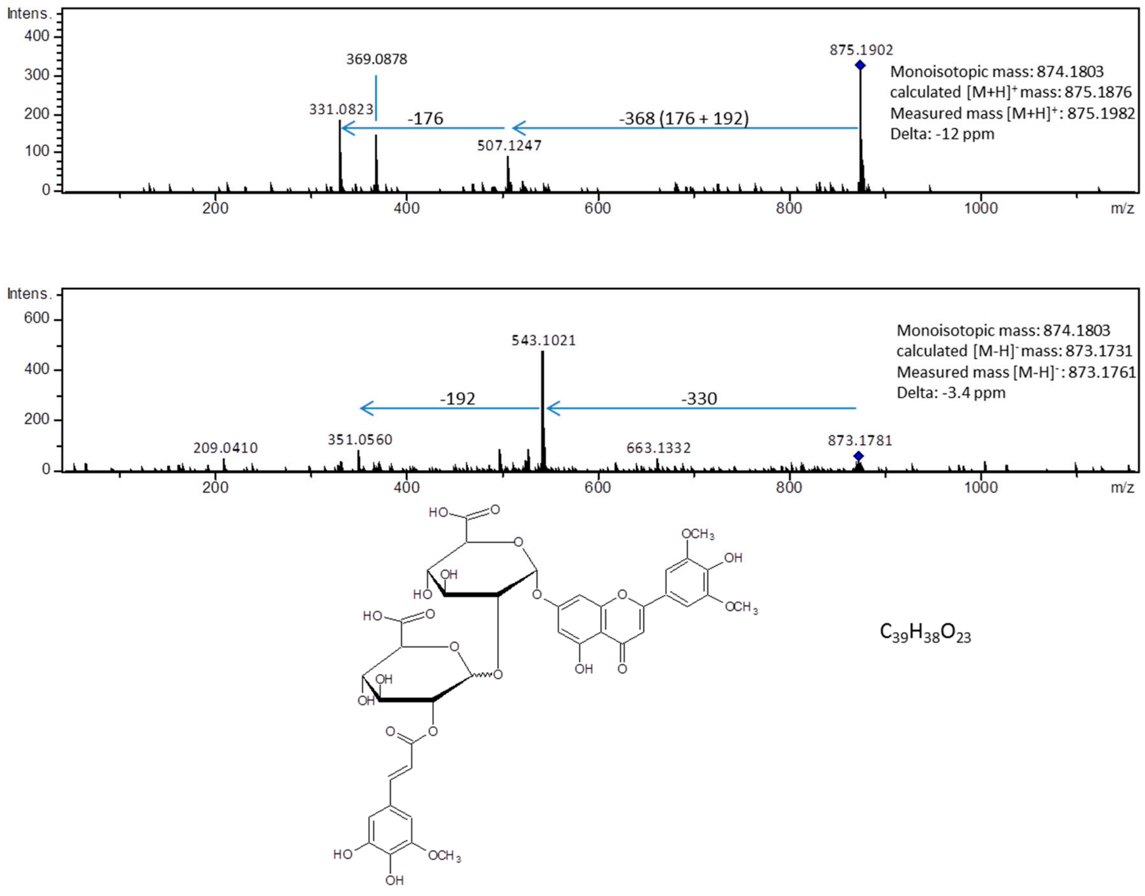

Figure S23. Tricin 7-*O*-[2′-*O*-5-hydroxyferuloyl-glucuronopyranosyl-(1-2)-*O*-glucuronopyranoside].

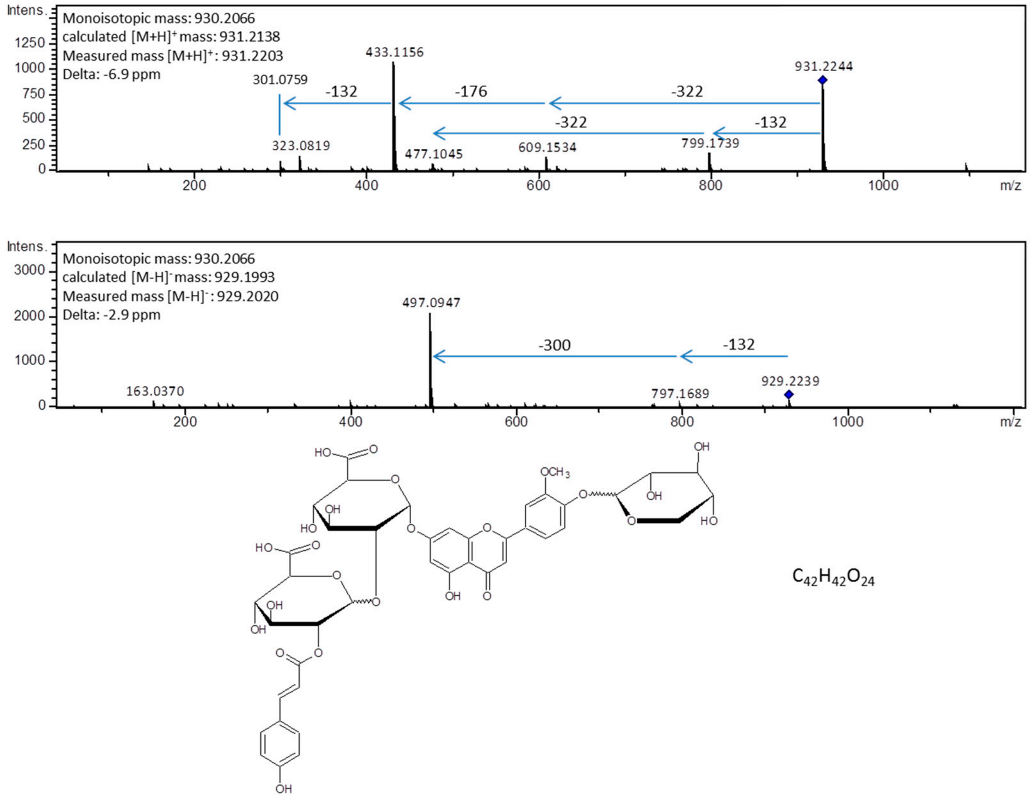

Figure S24. Chrysoeriol 4′-*O*-xylopyranosyl-7-*O*-[2′-*O*-coumaroyl-glucuronopyranosyl-(1-2)-*O*-glucuronopyranoside].

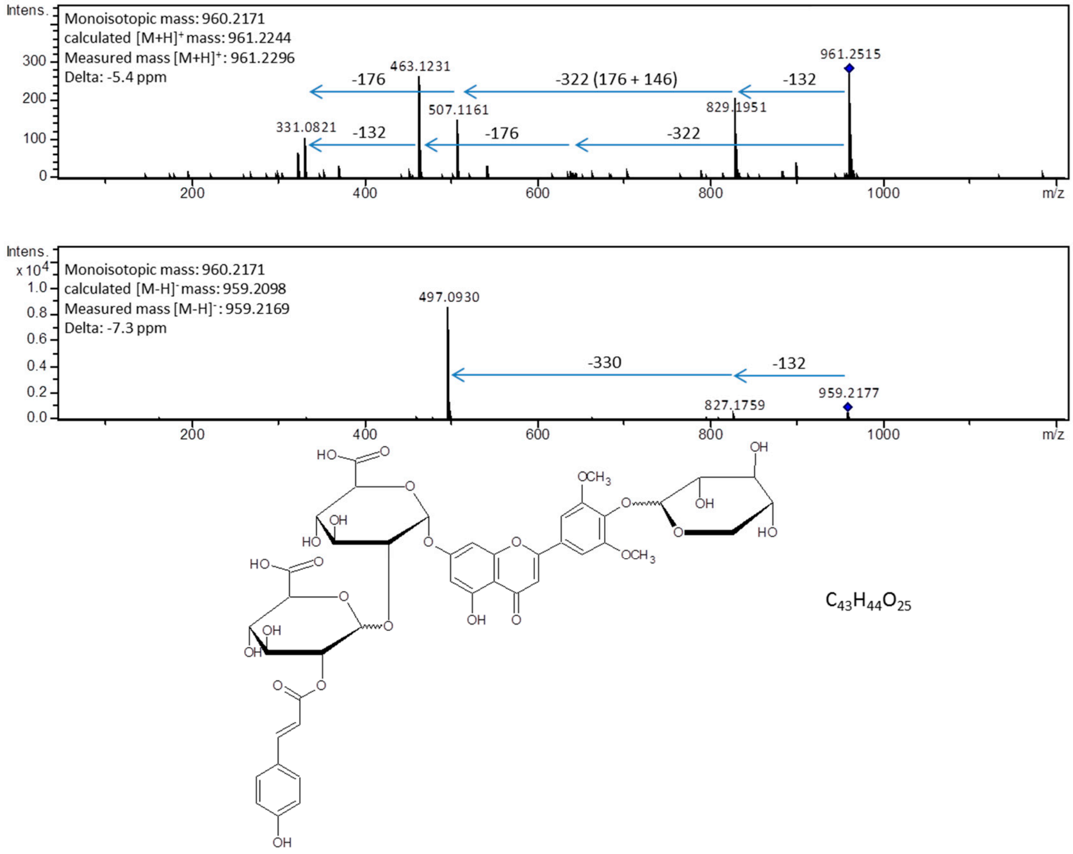

Figure S25. Tricin 4′-*O*-xylopyranosyl-7-*O*-[2'-*O*-coumaroyl-glucuronopyranosyl-(1-2)-*O*-glucuronopyranoside].

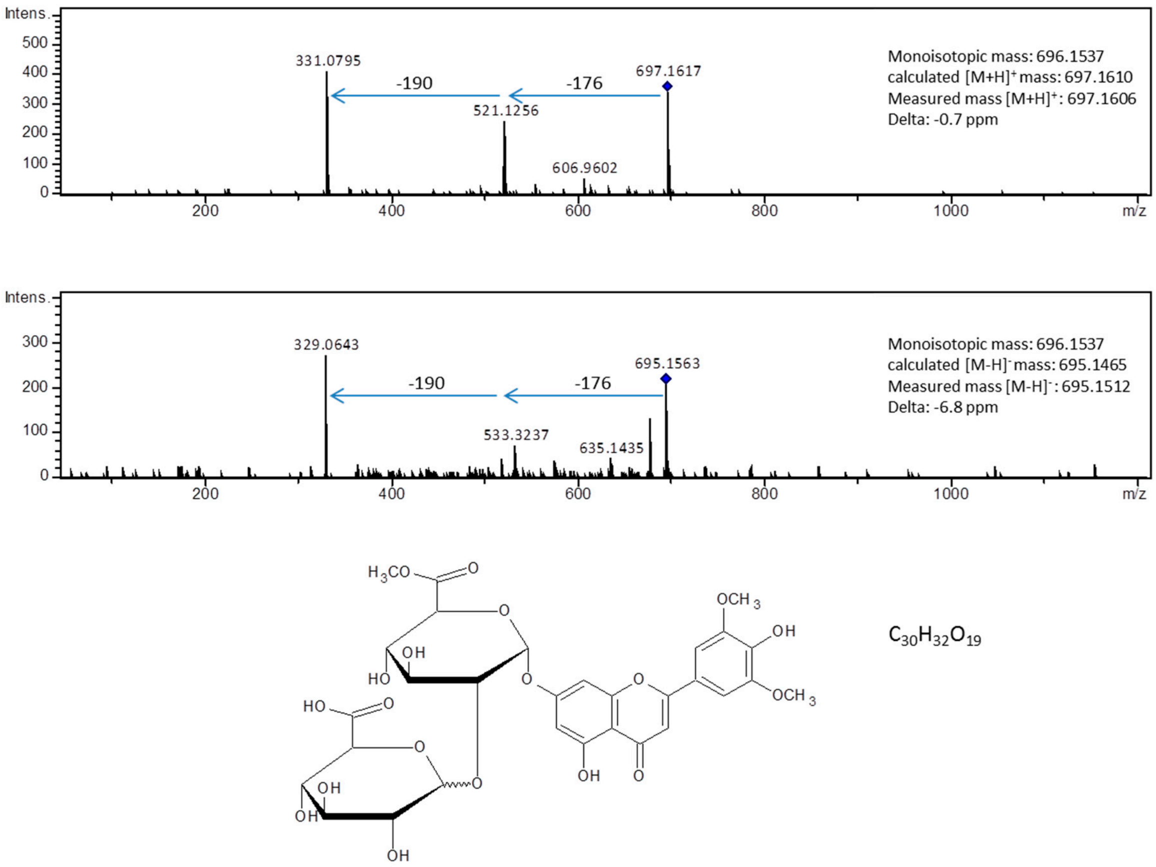

Figure S26. Tricin 7-*O*-[glucuronopyranosyl-(1-2)-*O*-methyloglucuronopyranoside].

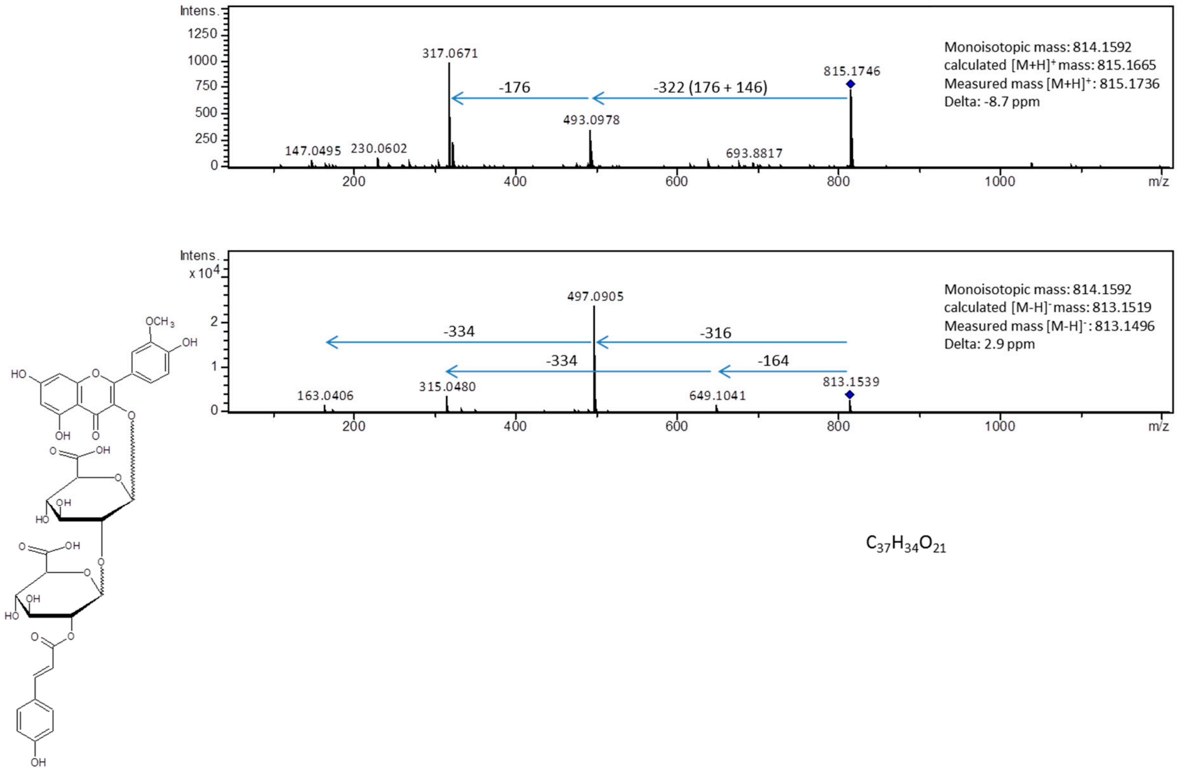

Figure S27. Isorhamnetin 3-*O*-[2′-*O*-coumaroyl-glucuronopyranosyl-(1-2)-*O*-glucuronopyranoside].

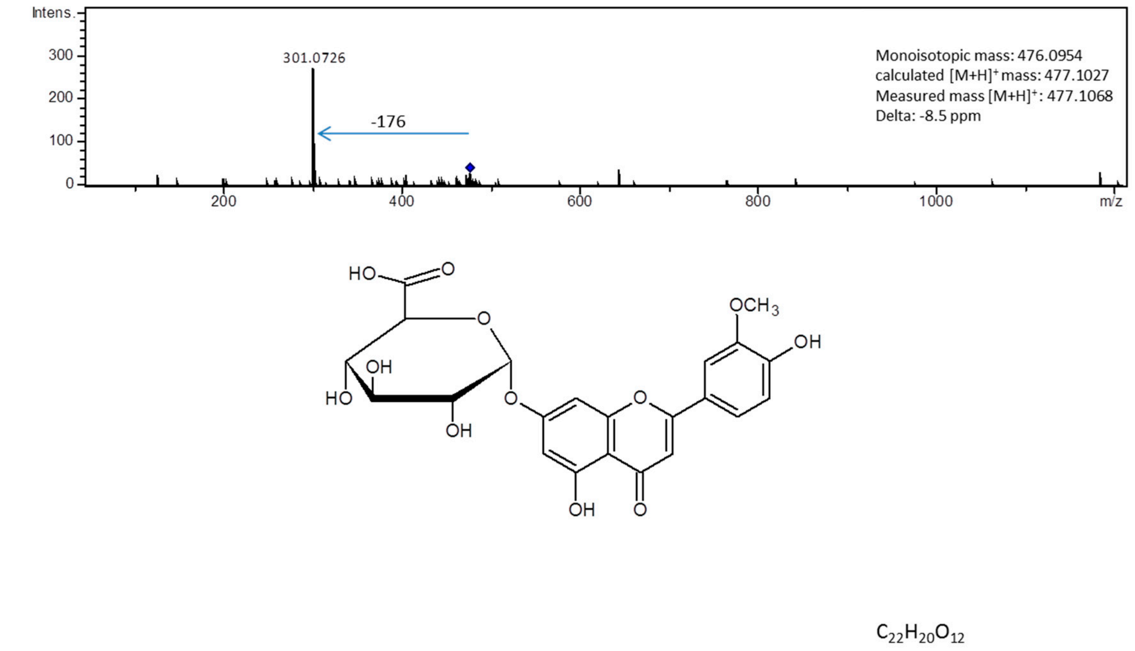

Figure S28. Chrysoeriol 7-*O*-glucuronopyranoside.

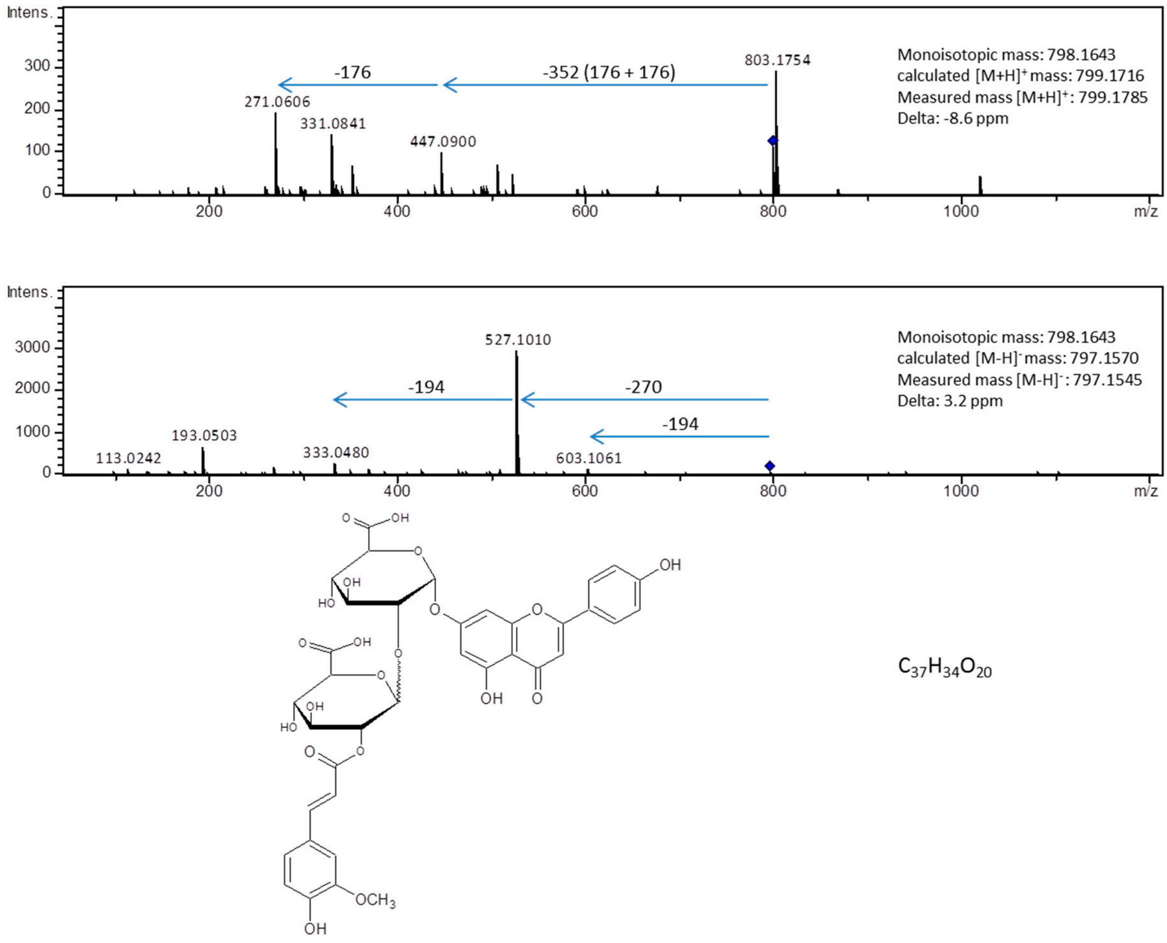

Figure S29. Apigenin 7-*O*-[2′-*O*-feruloyl-glucuronopyranosyl-(1-2)-*O*-glucuronopyranoside].

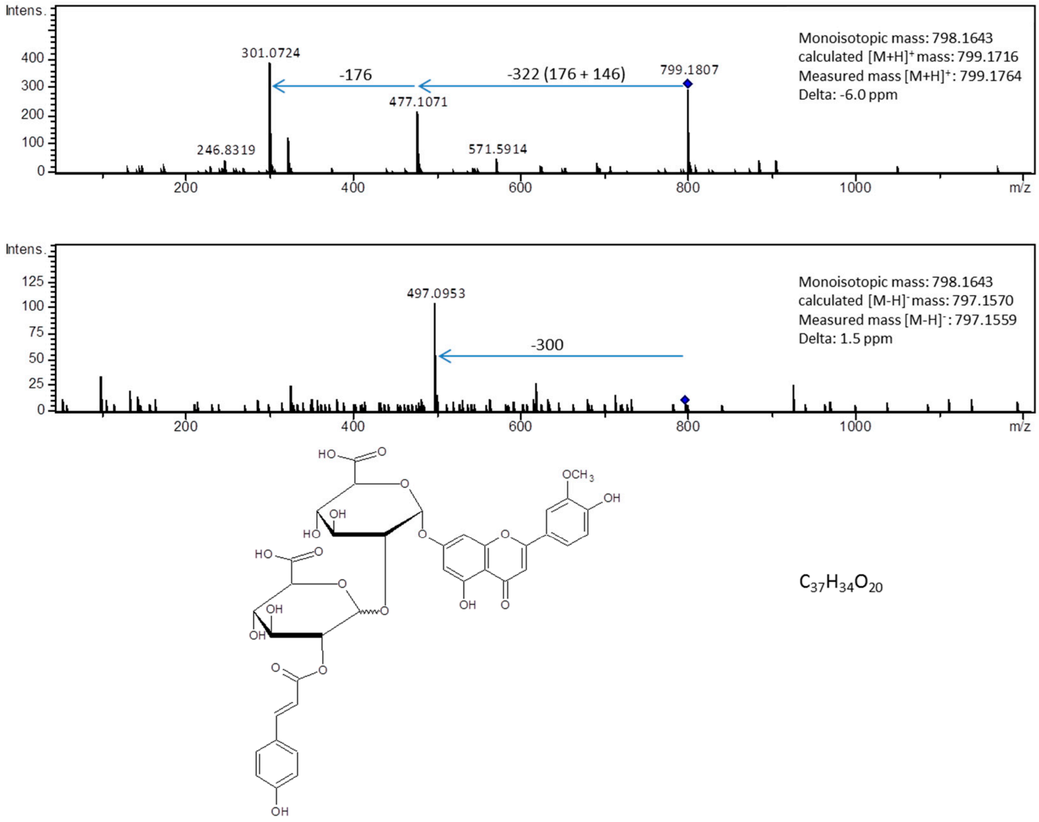

Figure S30. Chrysoeriol 7-*O*-[2′-*O*-coumaroyl-glucuronopyranosyl-(1-2)-*O*-glucuronopyranoside].

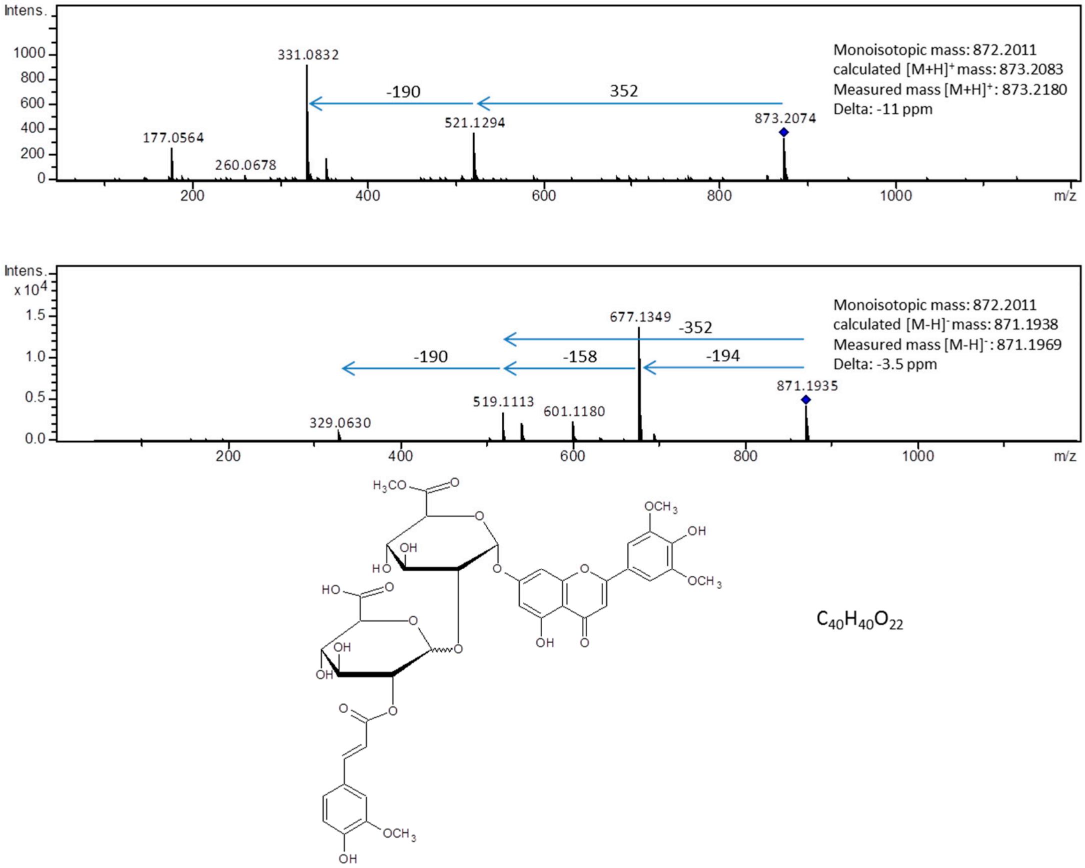

Figure S31. Tricin 7-*O*-[2′-*O*-coumaroyl-glucuronopyranosyl-(1-2)-*O*-methyloglucuronopyranoside].

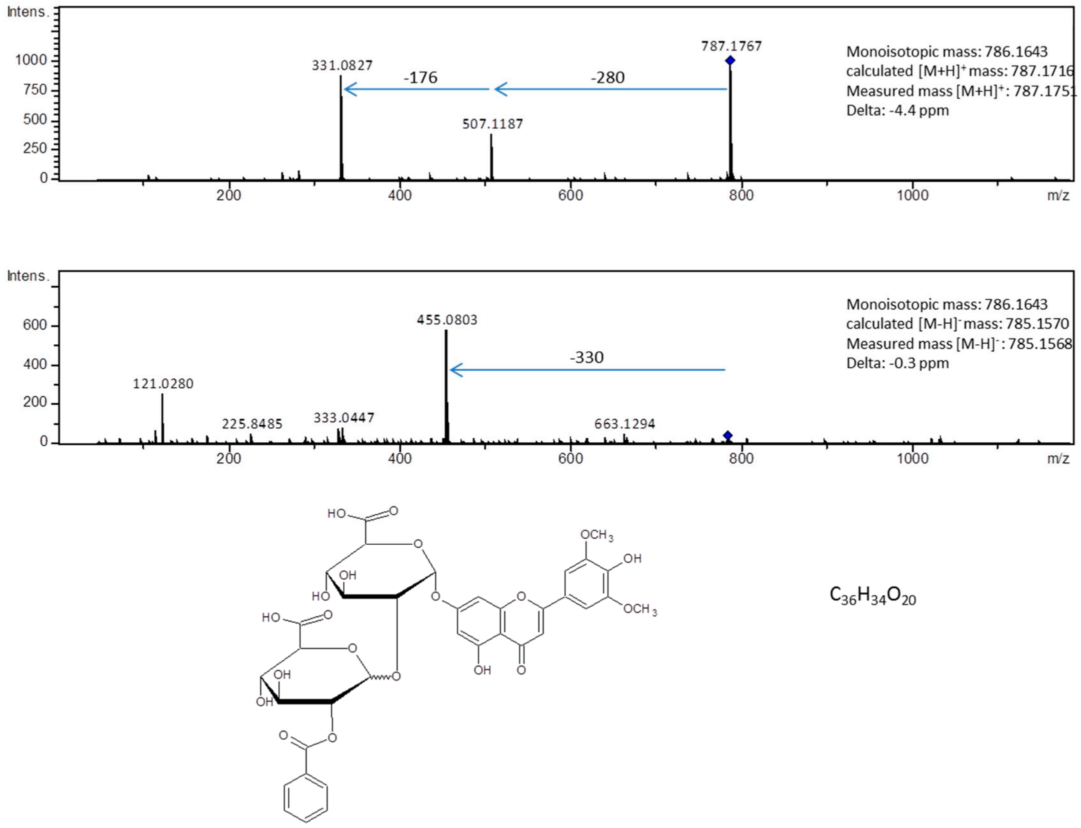

Figure S32. Tricin 7-*O*-[2′-*O*-benzoyl-glucuronopyranosyl-(1-2)-*O*-glucuronopyranoside].

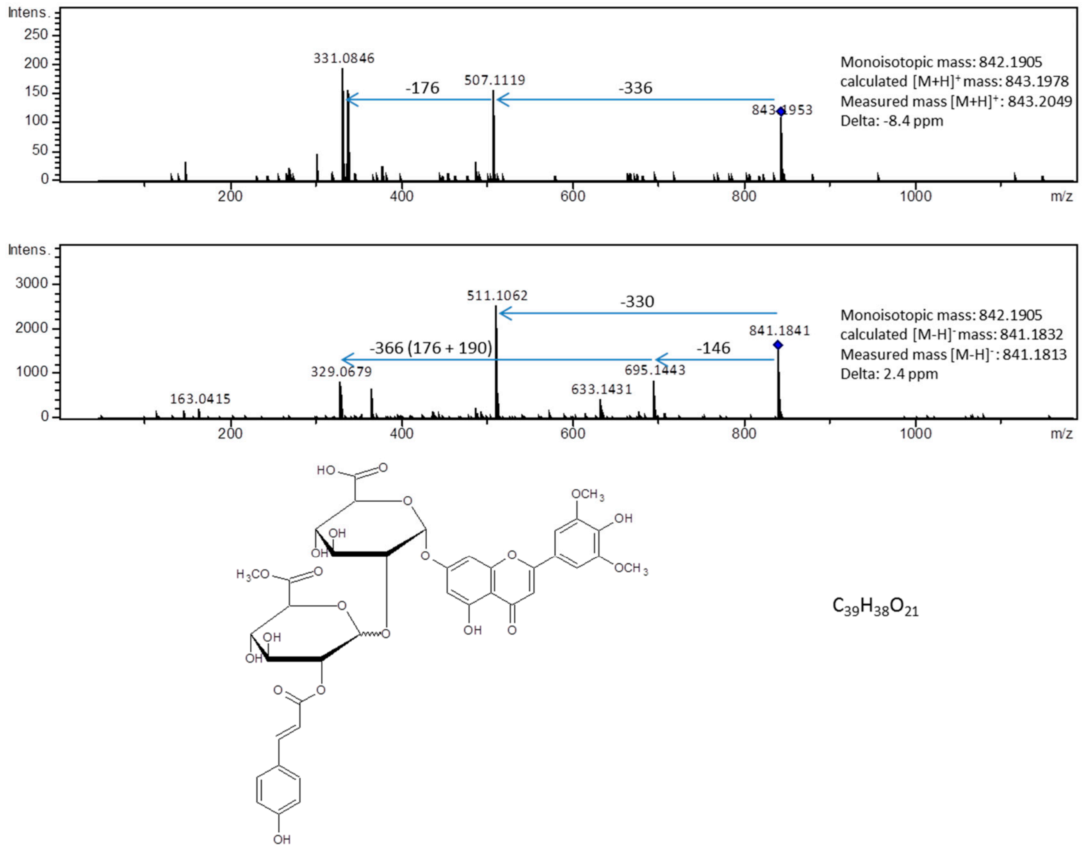

Figure S33. Tricin 7-*O*-[2′-*O*-coumaroyl-methyloglucuronopyranosyl-(1-2)-*O*-glucuronopyranoside].

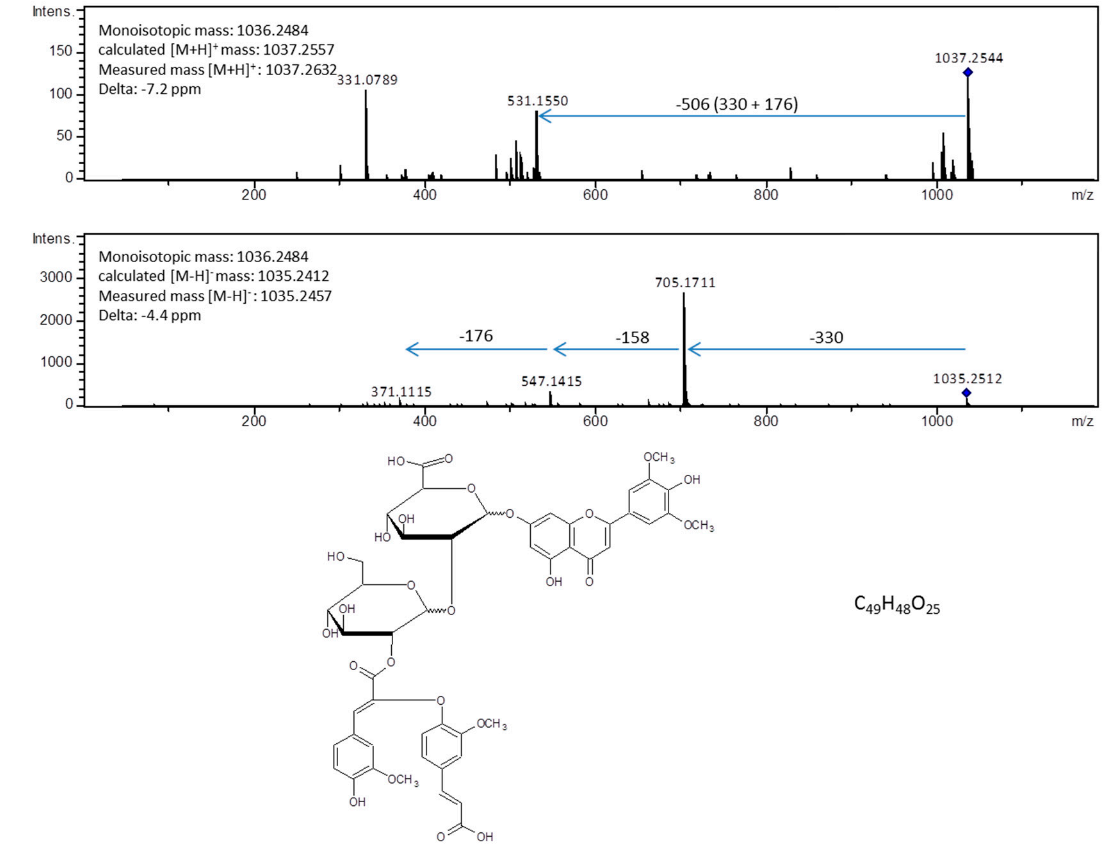

Figure S34. Tricin 7-*O*-[2′-*O*-dehydrodiferuloyl-glucopyranosyl-(1-2)-*O*-glucuronopyranoside].

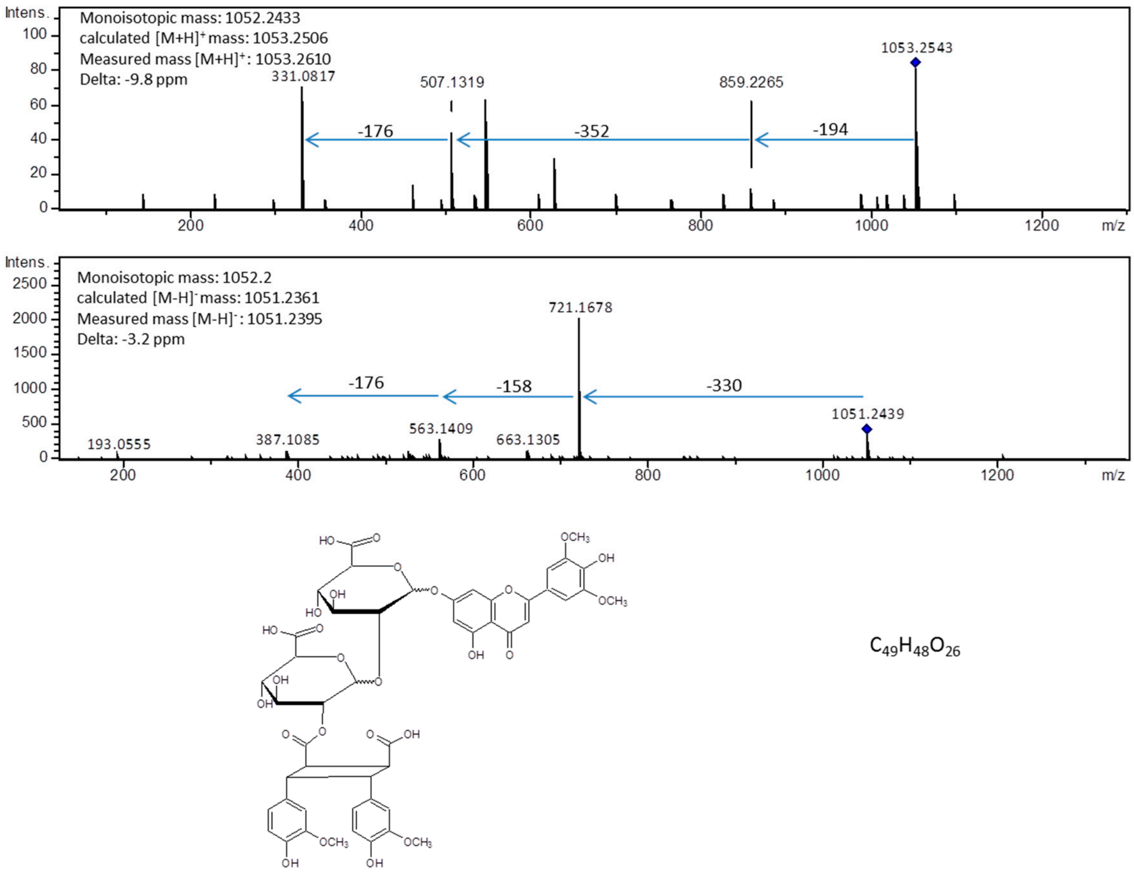

Figure S35. Tricin 7-*O*-[2′-*O*-dehydrodiferuloyl-glucuronopyranosyl-(1-2)-*O*-glucuronopyranoside].
